# Pixelating crop production: Consequences of methodological choices

**DOI:** 10.1371/journal.pone.0212281

**Published:** 2019-02-19

**Authors:** Alison K. B. Joglekar, Ulrike Wood-Sichra, Philip G. Pardey

**Affiliations:** 1 Department of Applied Economics, University of Minnesota, Saint Paul, Minnesota, United States of America; 2 International Food Policy Research Institute, Washington, D.C., United States of America; University of Maryland at College Park, UNITED STATES

## Abstract

Worldwide, crop production is intrinsically intertwined with biological, environmental and economic systems, all of which involve complex, inter-related and spatially-sensitive phenomena. Thus knowing the location of agriculture matters much for a host of reasons. There are several widely cited attempts to model the spatial pattern of crop production worldwide, not least by pixilating crop production statistics originally reported on an areal (administrative boundary) basis. However, these modeled measures have had little scrutiny regarding the robustness of their results to alternative data and modeling choices. Our research casts a critical eye over the nature and empirical plausibility of these types of datasets. To do so, we determine the sensitivity of the 2005 variant of the spatial production allocation model data series (SPAM2005) to eight methodological-cum-data choices in nine agriculturally-large and developmentally-variable countries: Brazil, China, Ethiopia, France, India, Indonesia, Nigeria, Turkey and the United States. We compare the original published estimates with those obtained from a series of robustness tests using various aggregations of the pixelized spatial production indicators (specifically, commodity-specific harvested area, production quantity and yield). Spatial similarity is empirically assessed using a pixel-level spatial similarity index (SSI). We find that the SPAM2005 estimates are most dependent on the degree of disaggregation of the underlying national and subnational production statistics. The results are also somewhat sensitive to the use of a simple spatial allocation method based solely on cropland proportions versus a cross-entropy allocation method, as well as the set of crops or crop aggregates being modeled, and are least sensitive to the inclusion of crude economic elements. Finally, we assess the spatial concordance between the SPAM2005 estimates of the area harvested of major crops in the United States and pixelated measures derived from remote-sensed data.

## Introduction

Where in the world agriculture occurs is consequential. Crop yields, for example, are the result of a complex and spatially sensitive set of interactions between climate, soil, crop variety and innumerable other crop management and input use choices made by farmers [1]. But we have comparatively little knowledge of precisely where (and how) crops are grown the world over. The diversity and spatial variation in cropping that stems from differences in agro-ecological conditions and market structures may not be adequately captured when using agricultural production statistics delineated by coarse administrative boundaries (e.g., countries or regions), such as those commonly reported by the Food and Agricultural Organization (FAO) or national statistical offices with whom they collaborate. In response, analysts and others have sought higher-resolution, pixelated data on crop production to better represent the spatially heterogeneous nature and impacts of agriculture.

Remote-sensed data, increasingly with global coverage, enables the gathering of timely and spatially-delineated data on cropping practices worldwide. However, highly detailed maps of cropland, crop acreage and crop performance are only available from a handful of national agricultural monitoring programs [2–3]. Similar data products with the same temporal and spatial resolutions are presently not available for most of the countries in the world. Beyond the considerable cost of acquiring and processing remote-sensed images worldwide, there are marked variations in cropping systems that complicate efforts to develop an integrated characterization system [4]. For instance, while maize production is ubiquitous, maize fields vary in size, climatological and edaphic attributes, seasonal performance, varietal choice and other crop management practices (e.g., timing and use of fertilizer or multiple-cropping), thus complicating efforts to develop comparable crop coverage maps for all countries. However, as analytical capabilities improve and costs fall, the use of remote-sensed imagery to develop detailed, reliable, spatially-calibrated maps of crop acreage and yield is bound to increase.

Meanwhile, other methods are being used to address the myriad of demands for pixilated crop production data, all of which entail procedures to “spatially downscale” or “spatially allocate” areal data reported as national or subnational aggregates or (in the case of yields) averages. You and Wood [5] identified three simple allocation methods, whereby hierarchical, areal crop production data (i.e., data at national versus regional versus local scales) are allocated to pixels within subordinate administrative areas in proportion to the distribution of total land area, cropland area, or biophysically suitable land for agriculture within each pixel. For example, one widely cited set of pixelated production estimates developed by Monfreda et al. [6] used pixelated cropland estimates reported by Ramunkutty et al. [7] to spatially allocate area data for each crop in proportion to the estimated share of total cropland within each pixel. Monfreda et al. [6] also assumed that the average yield for each administrative unit equaled the yield for each and every pixel within that unit, then (with some correction for cropping intensity) used these yield data in conjunction with the allocated area data to impute crop quantities for each pixel. Portmann et al. [8] took the Monfreda et al. [6] harvested area estimates and partitioned them further into irrigated and non-irrigated areas within each pixel.

A second, more complex method of spatially allocating areal data uses ancillary data on population density, crop suitability, irrigation and so forth to create a plausible, pixel-level sense of the geography of crop area, production and yield. This approach was first described by You and Wood [9], and subsequently used by Fischer et al. [10] and You et al. [11]. Anderson et al. [12] examined the spatial concordance of alternative circa 2000 estimates reported in Monfreda et al. [6], Portmann et al. [8], Fischer et al. [10] and You et al. [11] and found substantial differences.

Based on circa 2005 data obtained from You et al. [13], developed using their complex spatial production allocation method (dubbed SPAM), we conduct an in-depth assessment of the implications of using alternative data and modeling choices on the estimated landscape of crop production for 42 crops in nine countries. Our robustness assessment focuses on the empirical implications of eight methodological and data choices; specifically, the type of spatial allocation method, the crop coverage, the treatment of a “rest-of-crops” aggregate, the incorporation of a “crop suitability” data layer, the inclusion of rudimentary economic elements, and the administrative boundary details of the primary source statistics.

A spatial similarity index (SSI) index is used to assess the sensitivity of the SPAM2005 allocation method to each of the methodological-cum-data choices we studied. Each of the eight options has significant implications for the measured landscape of crop production, but some matter more than others. SPAM2005 is most sensitive to the spatial resolution of the underlying subnational crop production statistics, and moderately sensitive to the choice of the spatial allocation method used and the choice of crops or crop aggregates included. Finally, we assess the spatial concordance of harvested area from the original SPAM2005 estimates and variants thereof resulting from our robustness tests to the remote-sensed, pixilated data on U.S. cropping acreages reported by in the United States Department of Agriculture (USDA), National Agricultural Statistics Service’s (NASS) Cropland Data Layers (CDL) [14–16].

## Materials and methods

### Data

The data examined in this paper were sourced from HarvestChoice’s SPAM2005 v3r1 [13] global estimates of physical area, harvested area, production quantity and yield centered on the year 2005 for 42 crops and crop aggregates, and are available for download at www.mapspam.info. SPAM2005 disaggregates its estimates by four production systems (namely, irrigated, rainfed-high inputs, rainfed-low inputs and rainfed-subsistence). Our robustness assessments are based on the sum (for harvested area or production quantity) or area-weighted average (for yields) across these four production systems.

SPAM2005 estimates begin as informed priors on the pixel-level physical cropping area, which are developed in a pre-processing step using ancillary data on crop statistics, cropland, irrigated area, suitable area, population, crop prices and potential yields. These prior global 5 arc-minute pixelated estimates are then updated by way of a cross-entropy optimization model subject to several constraints to derive the *allocated* physical cropping area in each pixel. Using information on cropping intensities, production systems and potential yields, the allocated physical area is subsequently converted to estimates of allocated harvested area, production quantity and yield. The data and modeling methods used to create the SPAM2005 spatial crop production estimates are summarized in S1 Appendix in the Supporting Information, and are fully documented in Wood-Sichra et al. [17]. Our analysis focuses on nine agriculturally-important countries that represent a range of agro-ecologies, geographical regions, and per capita income classes (indicating, inter alia, various stages of economic development): namely Brazil, China including Taiwan (hereafter, China), Ethiopia, France, India, Indonesia, Nigeria, Turkey and the United States.

### Robustness scenarios

The estimates provided by SPAM2005 (or any similar spatial allocation model) are only as reliable as the methodology and data choices that underpin them. The data and methodological details we examine are summarized in Table 1, and the procedures we used to construct our eight robustness tests are discussed in more detail in S2 Appendix. With the exception of our test of the allocation method per se, each of the remaining robustness tests were performed by re-running SPAM2005 subject to the exclusion of a particular piece of information (e.g., 34 versus the 42 crops or crop aggregates in the reference SPAM2005 estimates, with and without a land suitability data layer or a market access layer, and so on). To test the empirical consequences of the allocation method per se, we compared the original SPAM2005 results (dubbed “complex method”) with those obtained using the Monfreda et al. [6] procedure (designated “simple method”) applied to the data elements underlying You et al. [13] (see S3 Appendix for details).

**Table 1:** Summary of robustness scenarios.

	Robustness Test	Original Description	Test Description
1	Allocation Method	Use complex method to disaggregate administrative-level statistics to pixels	Use simple method to disaggregate administrative-level statistics to pixels
2	Crop Choice	33 major crops and 9 crop aggregates	30 major crops and 4 crop aggregates
3	Remainder Allocation	Actively allocate rest-of-crops aggregate along with other 41 crops and crop aggregates	Passively assign remaining cropland after allocation to rest-of-crops aggregate
4	Crop Suitability	Allocated physical area by pixel, crop and production system constrained to not exceed the suitable area within the pixel with corresponding crop and production system	No suitability constraint in place during allocation process
Economic Suitability			
5	Market Access	Measure of market access varies by pixelated estimates of rural population density	No variation in market access measure
6	Crop Price	Global commodity prices vary by crop	No variation in global commodity prices (i.e., all prices are set to 1.0 I$/mt in model)
Underlying Statistics			
7	ADM0 Only	If available, production statistics collected at ADM2-level used before ADM1-level or ADM0-level	Only ADM0-level production statistics used
8	ADM1 Only	If available, production statistics collected at ADM2-level used before ADM1-level or ADM0-level	If available, production statistics collected at ADM1-level before ADM0-level

Throughout this paper the spatial unit of analysis for the areal data are statistical reporting units (SRUs). In keeping with Wood-Sichra et al. [17], SRUs constitute the most disaggregated geo-political unit for which the primary data are available for each country in our analysis. Fig A in S1 Appendix summarizes the spatial resolution of the crop-level source data at the ADM0- (national-level), ADM1- (subnational-level one) and ADM2-levels (subnational-level two). For Brazil, China, Ethiopia, India, Turkey and the United States the data were primarily compiled at an ADM2-level, although some reported crop coverage estimates in each country were only available at an ADM1-level. In the United States, there were counties with missing data for all crops except cotton. These data were predominately missing for non-disclosure reasons. In Indonesia and Nigeria, data were compiled for all crops at an ADM1-level. In France, most data were compiled at an ADM1-level; although data on eight crops were only available at the national-level. Robustness tests 1–6 in Table 1 maintain the original SRUs, while tests 7 and 8 are used to explicitly examine the empirical consequences of using data compiled only at the ADM1- or ADM0-level respectively.

## Results

The pixelized crop allocation implications of each methodological choice are evaluated by comparing various aggregations of the spatial distributions of production statistics from each of the robustness scenarios against the original (published) SPAM2005 estimates.

### The modeled presence or absence of production by pixel

A primary point of distinction among the different scenarios we assess is whether or not a crop is present or absent from a given pixel, irrespective of the magnitude of measured or modeled production. The upper half of Table 2 gives a country-level perspective on the spatial extent of maize within each of the nine listed countries. Column 2 reports the share of total cropland pixels within each country (reported in Column 1) where SPAM2005 reports non-zero maize production (cropland data was sourced from Fritz et al. [18]). Columns 3–10 report the share of pixels where maize is deemed to occur subject to each of the methodological-cum-data scenarios we examined. Thus, for example, SPAM2005 estimates that maize grew on 79.0 percent of the 66,224 cropped pixels in Brazil in 2005. This compares with almost all the cropped pixels (95.2 percent) if the simplified Monfreda et al. [6] allocation procedure was used, or just 72.0 percent if ADM1 (i.e., provincial-level) data were used instead of the ADM2 (municipality-level) data that underpins the majority of the original SPAM2005 estimates for Brazil.

**Table 2:** Comparison of non-zero maize production pixels between original and robustness test estimates.

				Robustness Tests							
								Economic Suitability		Underlying Statistics	
		(1)	(2)	(3)	(4)	(5)	(6)	(7)	(8)	(9)	(10)
Aggregation		Cropland Pixels	Original	Allocation Method	Crop Choice	Remainder Allocation	Crop Suitability	Market Access	Crop Price	ADM0 Only	ADM1 Only
		(count)	(percent)								
Country											
	Brazil	66,224	79	95.2	79.3	79	80.6	79.2	79.1	101.2	72
	China	67,572	73.6	77.2	73.3	73.7	74.6	73.5	73.5		69.4
	Ethiopia	4,723	69.4	84	69.6	68.8	80.5	69.4	69.4	65.3	68.1
	France	8,309	84.3	81.2	78.9	79.2	80.2	79.2	79.1	79.6	79.2
	India	37,049	71.6	74.2	71.7	72	75.5	71.9	72		32.6
	Indonesia	24,964	79.7	93.7	66.4	59.1	85	79.1	79.7	85.9	59.1
	Nigeria	10,269	91.7	92.8	92.6	90.3	92.3	91.1	91.7		91.7
	Turkey	11,966	69.5	85.6	66.9	67.8	81.1	69.5	69.5	73.1	70
	United States	78,995	64.8	64.4	64.6	64.8	65.6	64.8	64.8	79.1	76.5
Ethiopia Region											
	Addis Ababa	6	83.3	66.7	83.3	83.3	83.3	100	83.3	83.3	83.3
	Afar	65	100	92.3	98.5	100	100	100	100	96.9	104.6
	Amhara	1,361	60.3	82.1	63.3	57.6	81.9	60.3	60.4	67.2	67.2
	Benishangul-Gumuz	264	98.9	96.2	98.9	98.9	98.9	98.9	98.9	8.3	98.5
	Dire Dawa	11	100	100	100	100	100	100	100	81.8	100
	Gambella	59	71.2	67.8	71.2	71.2	71.2	71.2	71.2	11.9	71.2
	Harari	3	100	100	100	100	100	100	100	100	100
	Oromia	1,686	69.6	84	75.5	70.3	79.8	69.6	69.8	67	66.8
	SNNP	590	73.9	93.2	53.4	73.7	81.2	73.7	73.6	75.8	50.8
	Somali	168	47	53	47	47.6	54.2	47.6	47	51.8	82.1
	Tigray	510	74.5	82.5	72.9	74.5	76.1	74.5	74.5	77.6	68.2

Percentages over 100 percent are possible due to adjustments made to the cropland available to facilitate an entropy solution (see Wood-Sichra et al. 2016 for more details).

The lower half of Table 2 reports ADM1 level data for Ethiopia to illustrate the spatial sensitivity of the allocated presence or absence of maize per cropped pixel, again comparing the robustness test results against the original SPAM2005 estimates. S4 Appendix contains the same data for the other eight countries we studied. It reveals significant subnational differences in the modeled presence (and by implication absence) of maize throughout Ethiopia when comparing both across ADM1s for a given robustness test, and among robustness tests for a given ADM1. In some instances the differences are profound. For example, SPAM2005 estimates that 98.9 percent of the cropped pixels in the Benishangul-Gumuz region (located in western Ethiopia on the Sudanese border) grew maize in 2005, whereas allocating ADM0 (country-level) data puts the estimated share of cropped pixels growing maize in this region at just 8.3 percent.

Likewise, SPAM2005 estimates that 71.2 percent of the cropped pixels in the Gambella region (also located on the border region of western Ethiopia) grew maize, compared with just 11.9 percent of the region’s cropped pixels if the allocation process were initialized with just country-level data. These simulation results suggest that the crop suitability layer conditioning the spatial allocation process may cluster country-level data into highly localized pixels when unconstrained by more disaggregated ADM2 data. The ADM2 data implicitly incorporate many attributes (e.g., farmer choice and market access) into the spatial allocation process that go well beyond the climate and edaphic suitability attributes embodied in the Fischer et al. [10] crop suitability layer used by SPAM2005.

Consequently, there are significant spatial discrepancies in the pixilated presence or absence of production within a given SRU depending on the methodological-cum-data choices used in the spatial allocation procedure; discrepancies that tend to be magnified when the allocation procedure is primed with areal data from countries with large subnational administrative units like Ethiopia (where the average ADM2 unit is 13.1 square kilometers) versus countries like Brazil (where the average ADM2 unit is just 1.5 square kilometers). These spatial sensitivities point to the need for caution when interpreting the results of studies such as Franch et al. [19], Hutabarat et al. [20] and Johnson et al. [21] that use SPAM2005 data in their simulations taking this estimate of the geographical footprint of crop production at face value. Beddow et al.’s [1] results highlight the sensitivity of production and productivity assessments to the (changing) spatial footprint of crop production.

### Spatial evaluation criteria

A binary evaluation (presence versus absence) of crop production provides an important, first-cut perspective on the implications of different methodological and data choices on the modeled distribution of crop production, but there is added value in a more nuanced, spatially-explicit assessment of alternative spatial allocation approaches. The number of pixels in our nine-country sensitivity assessment is large; a total of 308,558 pixels. To develop a summary sense of the spatial implications of alternative data and modeling choices on the landscape of production we use a spatial similarity index (*SSI*). *SSI*_*i*_ is only calculated in those instances where pixel *i* has non-zero production for at least one of the modeled scenarios being compared. Additionally, we assessed similarity using the root mean squared errors (RMSEs) from an OLS regression of the robustness scenario estimates on the original estimates for each pixel *i* = 1 to *n*, but opted not to use this metric since RMSEs are highly sensitive to outliers and dissimilarity is not bounded (i.e., complete distinctiveness cannot be measured).

Our *SSI*_*i*_, based on Hangen-Zanker [22], measures the similarity of modeled production reported in pixel *i* estimated using one of our alternative methodological-cum-data choices, *a*_*i*,_ relative to modeled production for that same pixel taken from the original SPAM2005 data set, *b*_*i*,_. By construction, *SSI*_*i*_ ranges from 0 (entirely distinct) to 1 (identical), and is calculated between each pair of corresponding pixels (*a*_*i*,_ and *b*_*i*,_) using the following similarity function:

$$SSI_{i}=1-\frac{\left|a_{i}-b_{i}\right|}{\max\left(a_{i},b_{i}\right)}.$$ (1)

*SSI*_*i*_ can be used to examine differences on a pixel-by-pixel basis or summed across pixels at varying spatial scales (e.g., ADM 0, 1 or 2 levels of aggregation) or across pixel-crop combinations to provide a summary assessment of the relative consequences of alternative methodological-cum-data choices on the spatial distribution of production. Avitabile et al. [23] used this same approach to assess the spatial similarity of forest biomass throughout Uganda.

A raw pixel-wise comparison between the original and robustness scenario estimates can result in an exaggerated perception of spatial differences in crop production estimates, especially if the robustness scenario indicates an absence of production within a pixel whereas the original SPAM2005 estimate indicates some, albeit perhaps even minimal, production, within that same pixel (or vice versa). To address this problem, we recalculated each pixel value based upon a linear combination of values within a defined neighborhood of that pixel for each of the country-, crop-, and production system-specific estimates. Similar to Anderson et al. [12], we used Gaussian-based focal weights (or kernel files, see ESRI [24]) to account for the potential influence of neighboring pixels. But in contrast to Anderson et al. [12], given the comparatively large (5 arc-minute) spatial resolution of the SPAM2005 pixels, we opted to report the spatially averaged results from using focal weights with a 1-pixel radius and 0.33 standard deviation for the remainder of this analysis. Anderson et al. [12] used a 12-pixel radius and 3 standard deviation filter for their analysis on estimates with a similar 5 arc-minute resolution. We opted to use a radius that is three times the standard deviation (rather than four times the standard deviation) because 99.7 percent of the total integral of an infinite Gaussian filter falls within a radius of three standard deviations, and a finite choice larger than three standard deviations would unduly distort the shape of the Gaussian curve. Additionally, in our sample, the 5 arc-minute pixels with estimated crop production range in size from 2,774 hectares to 8,548 hectares, so the assumption that an individual 5 arc-minute pixel is affected by all of the neighboring pixels within a 12 pixel radius (which results in a total of 440 neighboring pixels versus our choice of 4) seems far-fetched. The implications of using alternative focal weights are presented in S5 Appendix, but none that we tried changed the qualitative nature of our results.

### Methodological choice sensitivity

To examine the country-level effects of methodological choices on SPAM2005, we first average each pixel-level *SSI*_*i*_ to a crop-level *j* indicator by summing and then averaging across each of the pixels *i* = 1 to *n* for a given country *k*:

$$SSI_{jk}=\frac{1}{n}\sum_{i=1}^{n}SSI_{ijk}.$$ (2)

Heatmaps of these crop-level *SSIs* are presented in S6 Appendix for each of the nine countries we examined. The spatial implications of the alternative methodological-cum-data choices we assessed vary markedly by crop and by country, but have less of a consequence for any of the production indicators (i.e., crop area, output or yield) for a particular crop-country combination. For example, while the pattern of *SSI* values for Brazil (S6 Appendix, Fig A) varies according to the modeling choices made, for a given modeling choice the *SSI* values are reasonably consistent across all 33 crops whose estimated spatial pattern of production was being assessed. While China’s *SSI* values (S6 Appendix, Fig B) are also sensitive to modeling choices (but in ways that are different from Brazil), in contrast to Brazil, China’s *SSI* values for a given modeling choice are also sensitive to the crop (in this instance 37 in total) under consideration.

**Fig 1:**
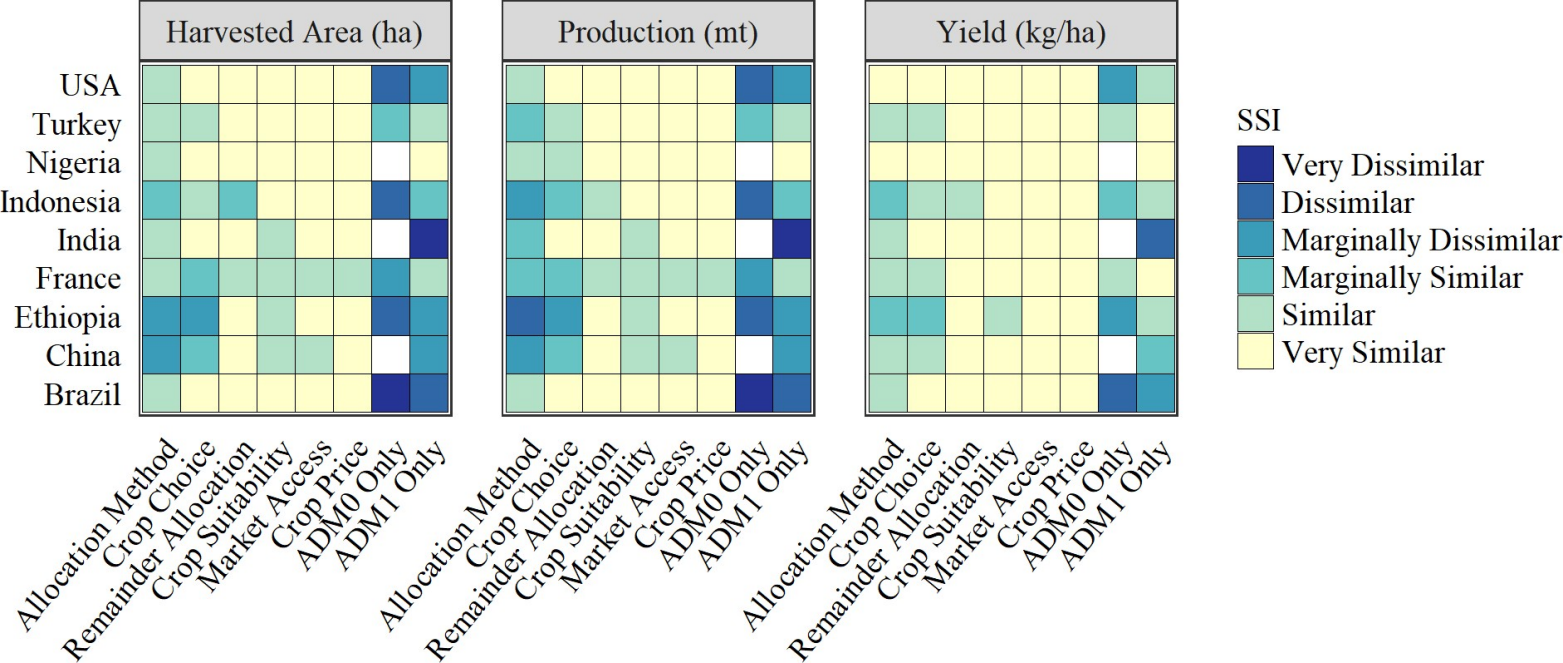
Spatial sensitivity of production to each robustness run relative to original estimates.

Fig 1 provides a summary, on-average, sense of the implications of modeling choices for each of the nine countries in our assessment. Here we present a weighted average of the crop-level *SSI*s ($SSI_{jk_{0}}$) for each country (revealed in S6 Appendix, Figs A to Fig 1), using crop-level harvested-area ($CropH_{jk_{0}}$) as the weight to reflect the relative spatial importance of each crop *j* in each country *k*:

$$SSI_{k}=\sum_{j=1}^{J}\left(SSI_{jk_{0}}\times \frac{\sum_{i=1}^{n}{CropH}_{ijk}}{\sum_{j=1}^{J}\sum_{i=1}^{n}{CropH}_{ijk}}\right).$$ (3)

A dark blue panel indicates a comparatively low pixel-by-pixel concordance in the spatial pattern of production for a given country-modeling choice combination, averaged across all crops. Increasingly lighter colored panels indicate increasingly higher pixel-by-pixel concordance in the location of crop production, on average. The general indication is that the spatial pattern of crop yields is less sensitive to methodological-cum-data choices than either harvested area or the quantity of crop production, irrespective of the country under consideration.

That said, the estimated pixilated pattern of output, area and, to some extent even, yields, tends to be more sensitive, and for some countries even quite sensitive, to the spatial resolution of the original tabulated data used to prime the SPAM2005 spatial allocation (maximum entropy) procedure. Notably, the *SSI*s in Fig 1 indicate that when the SPAM2005 procedure was primed with ADM0 (country-level) data, the resulting pixilated pattern of harvest area and production deviate markedly from the original SPAM2005 estimates. Similarly, using AMD1 data to prime the spatial allocation algorithm for Brazil and India leads to pixelated production estimates that do not concord closely with the original SPAM2005 estimates. In both instances, i.e., where either ADM0- (country) or ADM1- (first subnational) level data primed the allocation procedure, it was the countries with larger geographical crop footprints that tended to result in estimated spatial patterns of production that deviated the most from the original SPAM2005 estimates. For example, among the nine countries studied, Nigeria is one of the geographically smallest countries studied (0.91 million km^2^) compared with Brazil, which was among the largest (8.48 million km^2^, see S1 Appendix). Relying only on ADM1 harvested area data rather than much more granular ADM2 data to prime the spatial allocation procedure led to a large *SSI* value (0.998 for harvested area, thus spatially similar) for Nigeria versus a low *SSI* value (0.241 for harvested area, thus spatially dissimilar) for Brazil. India is an obvious anomaly to this generalization for reasons that we are unable to identify.

**Fig 2:**
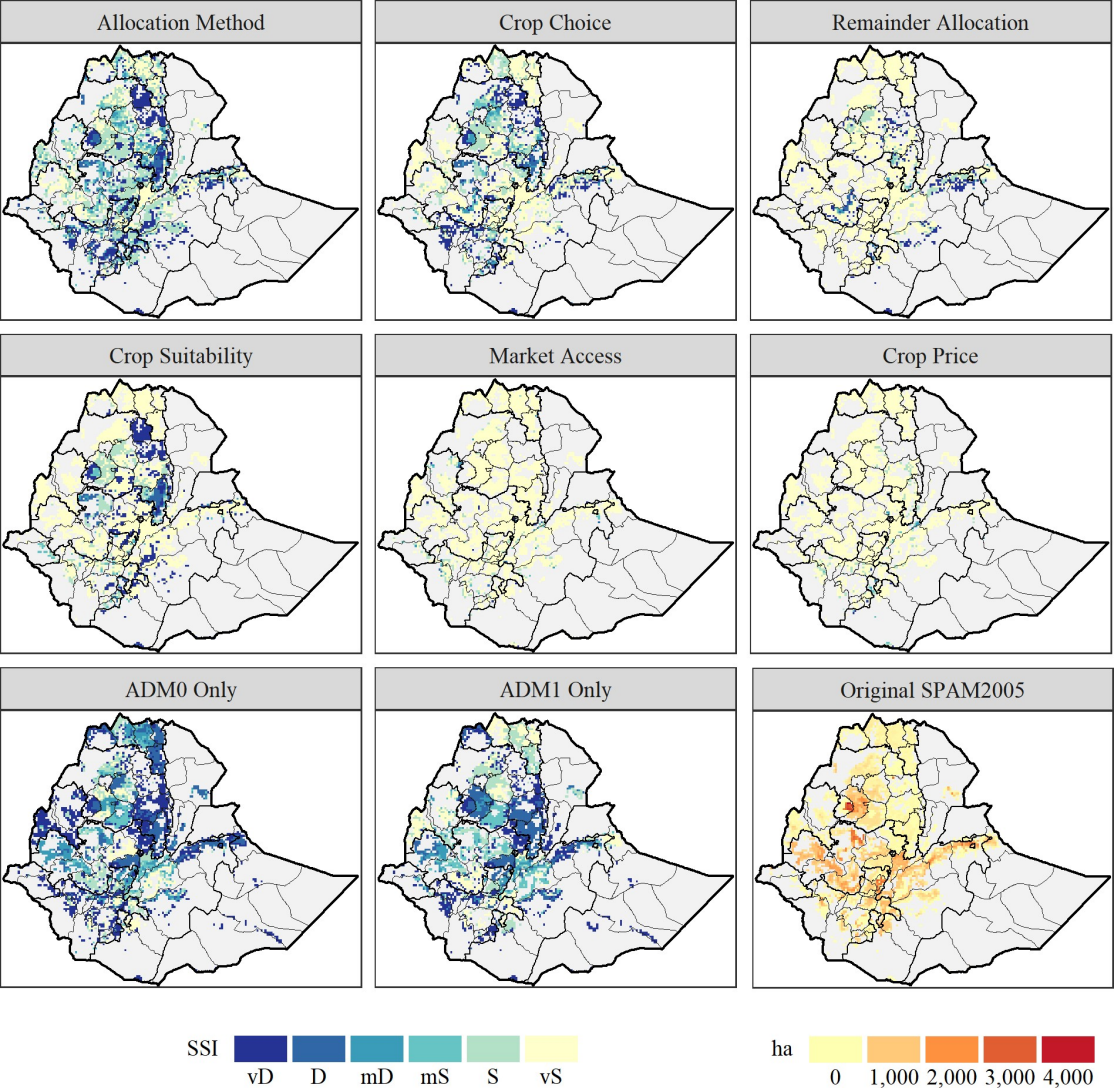
Spatial similarity index (SSI) for maize harvested area (ha) in Ethiopia, by robustness run. vD–Very Dissimilar; D–Dissimilar; mD–Marginally Dissimilar; mS–Marginally Similar; S–Similar; vS–Very Similar. *SSI* is calculated between each robustness run and original estimates. Areas not under cultivation are shaded light grey. The panel in the bottom right of the figure plots the original SPAM2005 estimates of harvested area.

Across the methodological variants we examined, Ethiopia and France exhibited the most sensitivity. To illustrate the locally variable nature of this sensitivity, Fig 2 gives a mapped representation of the pixel-level *SSI*s across methodological-cum-data choices along with the original SPAM2005 harvested area estimates for maize in Ethiopia. These sensitivity levels in Ethiopia and France may be due to several factors. Similar to the discussion above, for example, the average geographical sizes of the SRUs priming the crop allocations in both countries are relatively large; 13,140 km^2^ for ADM2 units in Ethiopia, and 24,960 km^2^ for ADM1 units in France. Imposing less geographical constraint on the location of production in the priors used to prime the allocation process increases the spatial degrees of freedom for the subsequent allocation algorithm, thus opening up the prospects of larger variation among alternative allocation procedures in the modeled location of production. Average administrative unit sizes are presented for each country in S1 Appendix.

In addition to the relatively large size of Ethiopia’s SRUs, another somewhat unique attribute (in the context of this study) is that the area weights used to form the country-level *SSI*s are heavily influenced by a crop aggregate, which consists largely of teff. FAO [25] does not report teff data per se, but rather includes the crop in their *cereals nes* (not elsewhere stated) aggregate. In Ethiopia, cereals nes account for 98.6 percent of the total area harvested in the *other cereals* category (with the residual 1.4 percent of that area in oats). The *other cereals* category, representing crops such as rye, buckwheat, quinoa and other minor crops in addition to oats, accounts for just 23.4 percent of the total area harvested in 2004/2006, and 12.3 percent of the total production quantity across all crops included in SPAM2005, thus indicating teff’s importance for Ethiopian agriculture. Optimizing the spatial allocation of crops using SPAM2005 requires the use of a substantial number of crop-specific parameters. However, if the optimization entails a “crop aggregate” there is no alternative but to use a set of proxy parameters (notionally representing the composite crop implied by the crop aggregate) to run the model. This may contribute to the apparent sensitivity of the Ethiopian results to methodological choices, compounded by the fact that in this case teff (a locally dominant, but internationally minor crop) has less than ideal data available.

While there are evident sensitivities to the spatial allocation of harvested area, quantity produced and crop yields associated with the granularity of the original data used to prime the allocation procedure, Fig 1 reveals much reduced sensitivity to the inclusion or exclusion of a (non-spatial) crop price element, the inclusion or exclusion of a market access layer, and the method used to allocate a rest-of-crops aggregate. Crop price was effectively removed from the spatial allocation procedure by setting all crop prices equal to one (see Wood-Sichra et al. [17]). The consequences of this methodological choice is that a crude potential revenue rather than potential quantity optimizing regime was implicit in the allocation procedure.

As a final sensitivity check, we examined the propensity of the allocation procedure to generate spatial clusters of harvested area, quantity and yield within the SRUs used to prime the allocation process. To test this proposition we calculated Moran’s I statistic (a measure of spatial autocorrelation) for each crop-country combination for each modeling scenario. As reported in S7 Appendix, we found no noticeable differences in the spatial autocorrelation of either harvested area, quantity produced or crop yield for the four crops (maize, rice, sorghum and wheat) we examined.

### Validation assessment

Pixelized estimates of crop production statistics represent a “plausible” accounting of the spatial structure of crop performance within a country, conditioned on a host of source data and measurement factors. To validate the SPAM2005 estimates, secondary data sets on crop production are needed, but finding statistics that have not already been used within the model is difficult, especially since these data have been shown to be important within SPAM2005. As an alternative, the present analysis utilizes the high-resolution CDL data products provided by the USDA, NASS. These layers delineate the major crop or land cover categories (e.g., wetlands or forest) within each 56 (year 2004) and 30 (years 2005 and 2006) meter pixel [14–16]. While accuracy assessment tables for the 2004, 2005 or 2006 CDL products are not published, Boryan et al. ([26] p. 342) state that “the quality of the CDL products was high with classification accuracies ranging in the low to mid-90% for major crops.”

The SPAM2005 estimates of harvested area in maize, soybeans, cotton, rice and wheat are compared with corresponding CDL estimates of physical area, averaged from 2004–2006 and aggregated to a 5 arc-minute grid resolution. When making direct comparisons between aggregated pixel-level information from CDL and SPAM, it is also worth noting that counting pixels and multiplying by the area of each pixel in the CDL will give a biased estimate of the aggregate acreage as compared with NASS official estimates because of Type 1 and 2 classification errors [26]. The differences between estimates of physical area and harvested area in the United States was trivial during this time period, due to limited instances of double-cropping [27]. Only states with complete coverage in all three years are used for our validation exercise: specifically, Illinois, Indiana, Iowa, Louisiana, Mississippi, Nebraska, North Dakota and Wisconsin. The crop-level *SSI*s—calculated using a state-level variant of Eq (2)—between the spatially-averaged physical area estimates reported by CDL and the spatially-averaged harvested area estimates reported by SPAM2005 are presented in Table 3 for the estimates from the original, allocation method, ADM0 only and ADM1 only scenarios.

**Table 3:** Spatial similarities between CDL and SPAM2005 crop area estimates.

		Spatial Similarity Indexes (*SSI*s)				
Robustness		Crop				
Scenario	State	Maize	Soybeans	Cotton	Rice	Wheat
Original	Illinois	0.70	0.72	0	0	0.42
	Indiana	0.71	0.69			0.50
	Iowa	0.82	0.82			0.21
	Louisiana	0.21	0.23	0.17	0.24	0.13
	Mississippi	0.20	0.24	0.18	0.10	0.19
	Nebraska	0.60	0.58	0		0.36
	North Dakota	0.34	0.36			0.52
	Wisconsin	0.34	0.33			0.22
Allocation Method	Illinois	0.70	0.72	0	0	0.43
	Indiana	0.70	0.68			0.52
	Iowa	0.82	0.82			0.21
	Louisiana	0.21	0.24	0.17	0.21	0.17
	Mississippi	0.21	0.25	0.19	0.10	0.25
	Nebraska	0.60	0.58	0		0.38
	North Dakota	0.39	0.37			0.51
	Wisconsin	0.37	0.38			0.26
ADM0 Only	Illinois	0.54	0.66	0.00	0.00	0.15
	Indiana	0.58	0.66	0	0	0.17
	Iowa	0.54	0.73	0	0	0.00
	Louisiana	0.17	0.22	0.19	0.15	0.14
	Mississippi	0.13	0.20	0.24	0.13	0.17
	Nebraska	0.38	0.40	0.00	0	0.19
	North Dakota	0.20	0.17		0	0.46
	Wisconsin	0.45	0.28		0	0.07
ADM1 Only	Illinois	0.63	0.68	0	0	0.30
	Indiana	0.66	0.67			0.44
	Iowa	0.77	0.79			0.23
	Louisiana	0.19	0.23	0.20	0.25	0.12
	Mississippi	0.19	0.24	0.21	0.19	0.16
	Nebraska	0.58	0.39	0		0.20
	North Dakota	0.28	0.14			0.40
	Wisconsin	0.36	0.26			0.18

Values reported as 0 are true zeros, while values of 0.00 indicate positive, but infinitesimally small values.

We examine five crops that represent major as well as minor crops within each of the eight states: namely, maize, soybean, cotton, rice and wheat. Maize is an important crop (i.e., grown on at least 30 percent of the total harvested area within a state) in Illinois, Indiana, Iowa, Nebraska and Wisconsin, while soybean is a similarly important crop in all eight states except North Dakota, where the major crop is wheat. In general, there is a reasonable to high spatial concordance between the CDL and SPAM2005 estimates for those crops that account for a significant share of the overall crop production within a state. For example, in Illinois, Indiana, Iowa and Nebraska, the CDL versus original SPAM2005 *SSI* values for the estimated area of maize and soybeans range from 0.58 to 0.82. However, the fact that a crop constitutes a large share of overall state production does not guarantee a strong concordance between the CDL and SPAM2005 estimates For example, in North Dakota, the spatial similarity for wheat (a crop that accounts for 44.6 percent of the state’s harvested area) between the original SPAM2005 and CDL estimates is just 0.52. Moreover, even comparatively minor crops can result in a reasonable degree of concordance between the CDL and SPAM2005 estimates. For example, the *SSI* for wheat in Indiana is 0.50 where wheat accounts for just 3.6 percent of the state’s harvested area. Regardless of the importance of each crop, the crop-level *SSI* values are uniformly low in Louisiana, Mississippi and Wisconsin.

One of the factors accounting for the discrepancies between the CDL and SPAM2005 spatial crop area estimates may be differences in the treatment of data due to disclosure concerns. As mentioned previously, in the agricultural census and related survey data that underpin the SPAM2005 estimates, NASS suppresses crop data in counties where there is a possibility of revealing information about an individual crop producer. There are no data suppression issues associated with the CDL estimates. Table 4 shows the share of counties for which the crop production data are not revealed in the NASS census and survey data. Rice and wheat statistics were withheld for a substantial number of counties. Likewise, crop statistics for a substantial number of counties in Louisiana, Mississippi and Wisconsin were undisclosed for all five of the crops included in our study.

**Table 4:** Share of counties with undisclosed crop production data, by state.

	Crop				
State	Maize	Soybeans	Cotton	Rice	Wheat
	(percent)				
Illinois	0.0	0.0			0.0
Indiana	0.0	0.0			28.3
Iowa	0.0	0.0			62.5
Louisiana	42.5	0.0	0.0	26.5	37.5
Mississippi	3.8	17.3	0.0	47.6	63.5
Nebraska	0.0	0.0			0.0
North Dakota	0.0	0.0			7.5
Wisconsin	11.4	12.9			38.6

We also examine the spatial concordance between the CDL estimates and estimates from three of our modeling choice scenarios—specifically, the allocation method, ADM0 only and ADM1 only. The *SSI*s indicate that the use of a simple versus a complex allocation method has little consequence for the observed relationship between the SPAM2005 versus CDL estimates (Table 3). However, variations in the spatial resolution of the source data are consequential for the concordance between the CDL and SPAM2005 estimates. In particular, the ADM0 only variant of SPAM2005 (i.e., using only country-level data to prime SPAM2005) tends to produce spatial patterns of production that do not concord closely with the CDL estimates, especially for the southern states and for maize and soybean in North Dakota. However, in line with findings discussed above, using more granular, ADM1 only (in this instance county-level) data to prime the SPAM2005 allocation method tends to improve the spatial concordance of the estimates vis-à-vis the CDL estimates, relative to when only ADM0 data were used.

**Fig 3:**
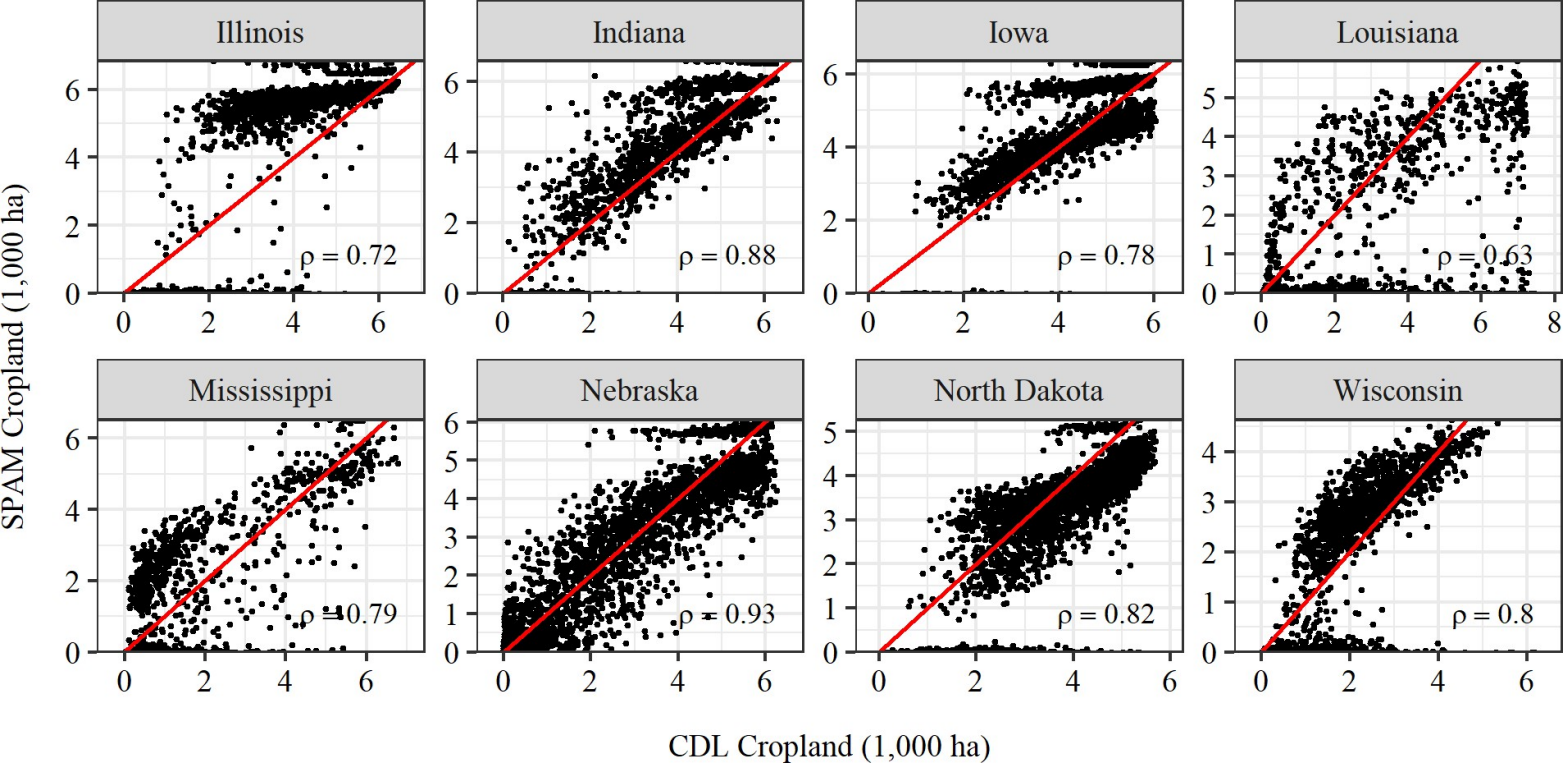
Pairwise comparison between CDL and SPAM2005 cropland estimates. Each plotted point represents the total cropland area within a pixel. The x-axis represents values inferred from CDL (by aggregating the area of pixels designated as a food crop) and the y-axis represents the total cropland area reported by Fritz et al. [18] and used by SPAM2005.

A significant portion of the crop-level differences between SPAM2005 and CDL estimates may lie in the differences in the spatial extent of the respective total land in crops layers underpinning (or in the case of CDL, implied by) these two sources. Fig 3 contains pairwise comparisons of the geography of the total land in crops within each state as reported by CDL and SPAM2005. There are reasonably strong, but by no means near perfect, positive correlations between the two pixel-level representations of cropland area for all states, with Nebraska reporting the highest correlation (ρ = 0.93). However, there are marked differences in the magnitude of some of the state-level cropland totals between the two sources. For example, in Louisiana the cropland aggregate implied by CDL is nearly twice as large as the cropland extent underpinning the SPAM2005 estimates, whereas in Iowa, Nebraska, Indiana and Mississippi, the two cropland extents differ by less than 10 percent.

## Discussion

The plausibility of the SPAM2005 (or any similarly derived) spatial crop production estimates is tied to the methodological decisions made in downscaling data from an areal to a pixelated representation. Remote-sensed, georeferenced crop data—although not without their own measurement issues—are still comparatively scarce, making it difficult to “independently” cross-validate downscaled spatial data such as SPAM2005. However, it is feasible to assess the sensitivity of the modeled SPAM2005 (and related) data products to systematic variations in the methodological choices made when generating these estimates. To that end, in this paper we quantitatively examined the relative influence of choices regarding the spatial allocation method, the crop coverage, the treatment of a “rest-of-crops” aggregate, the incorporation of a “crop suitability” data layer, the inclusion of rudimentary economic elements, and the administrative boundary details of the primary source statistics.

We show that the SPAM2005 estimates are most dependent on the degree of disaggregation of the underlying national and subnational statistics used to prime the allocation procedure. The results are moderately sensitive to the use of a simple allocation model—whereby areal, crop-specific production data are spatially allocated based solely on pixilated, aggregate cropland proportions—versus a cross-entropy allocation method. The results are also sensitive to the crop coverage included in the model. The influence of these methodological and primary data choices on crop harvested area, production and yields vary by country, crop and production statistic. Mis-characterizing the information used to prime the allocation model, such as the choice of cropping intensities or other ancillary data (most notably, assumed attributes pertaining to the crop aggregates), has the potential to introduce compounding errors into the spatial crop allocation procedure.

A particularly important, and somewhat unexpected result of our robustness analysis, was that removing (spatially invariant) crop prices and the measure of market access from the model had a relatively minor effect on the spatialized crop estimates (for most of the nine countries). While subsistence farming is prevalent in many parts of the world, one would expect that most of the production decisions made within global agriculture are intended to improve profitability. That relative output prices did not substantially influence the resulting spatial crop production estimates may be an artifact of the way prices are incorporated into the SPAM2005 model (i.e., via a quasi-revenue function rather than a (quasi-) gross margin or profit function). The SPAM2005 representation assumes that farmer crop choices are influenced by crop revenue relativities rather than something more akin to gross margin relativities. To calculate localized gross margins requires both local crop and input prices—see Joglekar [28] for an attempt to estimate spatialized fertilizer prices for Tanzania. However, it is more likely that using a spatially invariant (global average) crop price fails to reflect the local profitability relativities that affect farmers cropping choices.

Understanding the implications of these methodological choices within SPAM2005 can help researchers use this or similar data products with a better sense of their limitations. Our findings also point the way to potential refinement of this and related spatial production allocation models in the future. The finding that the SPAM2005 estimates are particularly sensitive to the quality and spatial precision of the underlying statistics used to prime the model is particularly pertinent. These and similar other source statistics underpin all the spatially allocated data products presently available, and so the veracity of all these other data products are likely to be subject to the same caveat.

## Supporting information

S1 Appendix SPAM2005 methodology. (DOCX)

S2 Appendix Robustness scenarios. (DOCX)

S3 Appendix Methodology outline for the proportional or alternative allocation method. (DOCX)

S4 Appendix Modeled presence or absence of pixels at subnational-level. (DOCX)

S5 Appendix Gaussian-based focal weights. (DOCX)

S6 Appendix Methodological choice sensitivity by country and crops. (DOCX)

S7 Appendix Spatial autocorrelation. (DOCX)

## References

[1] Beddow J, Hurley T, Pardey P, Alston J. Food security: Yield gap In:Van Alfen N, editor. Encyclopedia of agriculture and food systems, vol 3 San Diego, Elsevier; 2014 p. 352–365.

[2] GEO (Group on Earth Observations). Report from the workshop on developing a strategy for global agricultural monitoring in the framework of Group on Earth Observations (GEO), 16–18 July 2007, FAO, Rome. Geneva, Switzerland: Group on Earth Observations Available from: http://www.fao.org/gtos/igol/docs/meeting-reports/07-geo-ag0703-workshop-report-nov07.pdf. Cited September 2016.

[3] GEOGLAM (Group on Earth Observations Global Agricultural Monitoring). National Monitoring Systems. Available from: http://www.geoglam.org/index.php/en/national-systems. Cited December 2017.

[4] Pittman K, Hansen MC, Becker-Reshef I, Potapov PV, Justice CO. Estimating global cropland extent with multi-year MODIS data. Remote Sensing. 2010; 2: p. 1844–1863.

[5] You L, Wood S. An entropy approach to spatial disaggregation of agricultural production. Agricultural Systems. 2006; 90: p. 329–347.

[6] Monfreda C, Ramankutty N, Foley JA. Farming the planet 2: Geographic distribution of crop areas, yields, physiological types, and net primary production in the year 2000. Global Biogeochemical Cycles. 2008; 22: pp. 19.

[7] Ramankutty N, Evan AT, Monfreda C, Foley JA. Farming the planet 1: Geographic distribution of global agricultural lands in the year 2000. Global Biogeochemical Cycles. 2008; 22: pp. 19.

[8] Portmann FT, Siebert S, Döll P. MIRCA2000—Global monthly irrigated and rainfed crop areas around the year 2000: A new high resolution data set for agricultural and hydrological modeling. Global Biogeochemical Cycles. 2010; 24: pp. 24.

[9] You L, Wood S. Assessing the spatial distribution of crop production using a cross-entropy method. Washington, D.C.: International Food Policy Research Institute (IFPRI); 2004. EPTD Discussion Paper No. 126.

[10] Fischer G, Nachtergaele FO, Prieler S, Teixeira E, Tóth G, van Velthuizen H, et al Global Agro-Ecological Zones (GAEZ) Version 3.0. 2013 Available from: http://www.fao.org/nr/gaez/en/. Cited October 2014.

[11] You L, Wood S, Wood-Sichra U, Wu W. Generating global crop distribution maps: From census to grid. Agricultural Systems. 2014; 127: p. 53–60.

[12] Anderson W, You L, Wood S, Wood-Sichtra U, Wu W. An analysis of methodological and spatial differences in global cropping systems models and maps. Global Ecology and Biogeography. 2015; 24(2): p. 180–91.

[13] You L, Wood-Sichra U, Fritz S, Guo Z, See L, Koo J. Spatial Production Allocation Model (SPAM) 2005 version 3 release 1. Washington, D.C.: International Food Policy Research Institute (IFPRI) and St. Paul: International Science and Technology Practice and Policy (InSTePP) Center, University of Minnesota; 2017. HarvestChoice Data Product. Cited January 2017.

[14] Cropland Data Layer: 2004. Washington, D.C.: United States Department of Agriculture—National Agricultural Statistics Service (USDA-NASS). c2015. Available from: http://nassgeodata.gmu.edu/CropScape. Cited September 2015.

[15] Cropland Data Layer: 2005. Washington, D.C.: United States Department of Agriculture—National Agricultural Statistics Service(USDA-NASS). c2015. Available from: http://nassgeodata.gmu.edu/CropScape. Cited September 2015.

[16] Cropland Data Layer: 2006. Washington, D.C.: United States Department of Agriculture—National Agricultural Statistics Service (USDA-NASS). c2015. Available from: http://nassgeodata.gmu.edu/CropScape. Cited September 2015.

[17] Wood-Sichra U, Joglekar AB, You L. Spatial production allocation model (SPAM) 2005: Technical documentation. Washington, D.C: International Food Policy Research Institute (IFPRI) and St. Paul: International Science and Technology Practice and Policy (InSTePP) Center, University of Minnesota; 2016. HarvestChoice Working Paper.

[18] Fritz S, See L, McCallum I, You L, Bun A, Moltchanova E, et al Mapping global cropland and field size. Global Change Biology. 2015; 21: p. 1980–1992. Data available from https://www.geo-wiki.org/downloads/. 10.1111/gcb.12838 25640302

[19] Franch B, Vermote EF, Becker-Reshef I, Claverie M, Huang J, Zhang J, et al Improving the timeliness of winter wheat production forecast in the United States of America, Ukraine and China using MODIS data and NCAR growing degree day information. Remote Sensing of Environment. 2015; 161: p. 131–148.

[20] Hutabarat B, Setiyanto A, Kustiari R, Sulser TB. Conjecturing production, imports and consumption of horticulture in Indonesia in 2050: A GAMS simulation through changes in yields induced by climate change. Jurnal Agro Ekonomi. 2012; 30: pp. 23.

[21] Johnson M, Takeshima H, Gyimah-Brempong K. Assessing the potential and policy alternatives for achieving rice competitiveness and growth in Nigeria. Washington, D.C.: International Food Policy Research Institute (IFPRI); 2013. IFPRI Discussion Paper 01301.

[22] Hagen-Zanker A. Comparing continuous valued raster data: A cross disciplinary literature scan. Maastricht, The Netherlands: Research Institute for Knowledge Systems; 2005.

[23] Avitabile V, Herold M, Henry M, Schmullius C. Mapping biomass with remote sensing: A comparison of methods for the case study of Uganda. Carbon Balance and Management. 2011; 6(1): pp. 14 10.1186/1750-0680-6-14 21982054 PMC3198671

[24] ESRI (Environmental Systems Research Insitute). How focal statistics work. c2016. Available from: http://desktop.arcgis.com/en/arcmap/10.3/tools/spatial-analyst-toolbox/how-focal-statistics-works.htm. Cited June 2017.

[25] FAOSTAT Database Collection. Rome, Italy: Food and Agriculture Organization of the United Nations (FAO); c2017. Available from: http://www.fao.org/faostat/en/#data. Cited November 2017.

[26] Boryan C, Yang Z, Mueller R, Craig M. Monitoring US agriculture: the US Department of Agriculture, National Agricultural Statstics Service, Cropland Data Layer Program. Geocarto Interational. 2011; 26(5): p. 341–358.

[27] Borchers A, Truex-Powell E, Wallander S, Nickerson C. Multi-cropping practices: Recent trends in double copping Economic Information Bulletin Number 125. Washington, D.C.: Economic Research Service 2014.

[28] Joglekar A. The landscape of farming: An exploration of spatial bio-economic characterization approaches. Ph.D. Dissertation, University of Minnesota. 2015. Available from: http://hdl.handle.net/11299/193427.

