# Supplementary material for: Pixelating crop production: Consequences of methodological choices
Source: PLoS One. 2019 Feb 19;14(2):e0212281. doi: 10.1371/journal.pone.0212281 (PMC6380596; doi:10.1371/journal.pone.0212281)
Supplement: S1 Appendix — (DOCX) [file pone.0212281.s001.docx]

# S1 Appendix

The methodology underpinning SPAM2005 is extensively documented in Wood-Sichra et al. (2016), and summarized below.

## Data inputs

As our analysis reveals, a critical set of data for priming the SPAM2005 spatial allocation procedure used by You et al. (2017) to form pixilated crop production estimates are the national and subnational areal (tabulated) statistics on harvested area and yield for each of the 42 crops and crop aggregates studied. To form the 2005 estimates, areal data were collected from a variety of sources, including AgroMaps (FAO 2012a), CountrySTAT (FAO 2012c), Eurostat (European Commission 2012), national statistical offices, ministries of agriculture and household-level surveys. These data were then averaged over the 2004–2006 period and recalibrated so that the aggregated subnational data equaled the corresponding 2004–2006 FAOSTAT (FAO 2012d) national totals. Production statistics were collected at the most disaggregated *statistical reporting unit* (SRU) possible, be that within national (ADM0), subnational-level one (ADM1) or subnational-level two (ADM2) geo-political boundaries.

Table A presents descriptive statistics on the nine countries of interest. For all nine countries, data coverage (in terms of the availability of data for at least one of the 42 crops studied) was complete or nearly complete for all ADM0, ADM1 and ADM2 geo-political boundaries. The exceptions were France, Indonesia and Nigeria which reported no ADM2 statistics in the sources used by You et al. (2017). Fig A highlights the degree of missing source data for each crop by administrative level within a country (denoted as the share of administrative units with missing data for a crop). None of the six countries with ADM2 statistics available have complete coverage across all crops at this level information. In the United States, complete ADM2-level data are only

**Table A. Descriptive statistics on countries analyzed**

|  |  |  |  |  | Subnational Units | | | | | | | | | |
| --- | --- | --- | --- | --- | --- | --- | --- | --- | --- | --- | --- | --- | --- | --- |
|  |  |  |  |  |  | ADM1 | | | |  | ADM2 | | | |
| Country | Agro-ecology | Income | Country Size | Cropland |  | Count | Average Size | Average Crop Area | Data Coverage |  | Count | Average Size | Average Crop Area | Data Coverage |
|  |  |  | (million km2) | |  |  | (thousand km2) | | (percent) |  |  | (thousand km2) | | (percent) |
| Brazil | Temp/Trop | UM | 8.48 | 1.16 |  | 31 | 273.69 | 37.32 | 100.00 |  | 5,510 | 1.54 | 0.22 | 98.82 |
| China | Temp/Trop | UM | 9.42 | 1.97 |  | 38 | 247.86 | 61.70 | 100.00 |  | 2,430 | 3.88 | 0.83 | 98.27 |
| Ethiopia | Tropics | L | 1.13 | 0.16 |  | 11 | 102.86 | 14.50 | 100.00 |  | 86 | 13.14 | 2.07 | 89.61 |
| France | Temperate | H | 0.55 | 0.20 |  | 22 | 24.96 | 9.00 | 100.00 |  | 96 | 5.72 | 2.06 | - |
| India | Temp/Trop | LM | 2.98 | 2.18 |  | 34 | 87.63 | 62.35 | 100.00 |  | 576 | 5.17 | 3.67 | 98.42 |
| Indonesia | Tropics | LM | 1.89 | 0.52 |  | 33 | 57.23 | 16.69 | 100.00 |  | 443 | 4.28 | 1.21 | - |
| Nigeria | Tropics | LM | 0.91 | 0.54 |  | 37 | 24.57 | 14.65 | 100.00 |  | 547 | 1.66 | 1.03 | - |
| Turkey | Temperate | UM | 0.78 | 0.34 |  | 12 | 65.11 | 28.00 | 100.00 |  | 26 | 29.97 | 12.93 | 100.00 |
| USA | Temperate | H | 9.33 | 1.47 |  | 51 | 182.87 | 28.83 | 100.00 |  | 3,131 | 2.98 | 0.47 | 80.14 |

*Source:* Authors’ construction using data from You et al. (2017), the 2005 World Bank country and lending groups definitions: http://data.worldbank.org/about/country-and-lending-groups, and relevant 2006-year shapefiles from FAO (2014), GADM (2015) and European Commission (2008).

*Note*: ADM1 – administrative level one; ADM2 – administrative level two; H – High Income; UM – Upper Middle Income; LM – Lower Middle Income; L – Low Income. Area statistics were calculated from FAO GAUL shapefiles for Brazil, Ethiopia, France, India, Indonesia, Nigeria and the United States; GADM shapefiles for China and Taiwan; and Eurostat NUTS shapefiles for Turkey. Coverage statistics represent percentage of administrative units with statistics on at least one crop. If a crop is known to not be in production within a country (as opposed to missing data), it was included in the coverage statistic calculation.

**Fig A: Share of administrative units with missing crop production data, by administrative level and country**

**
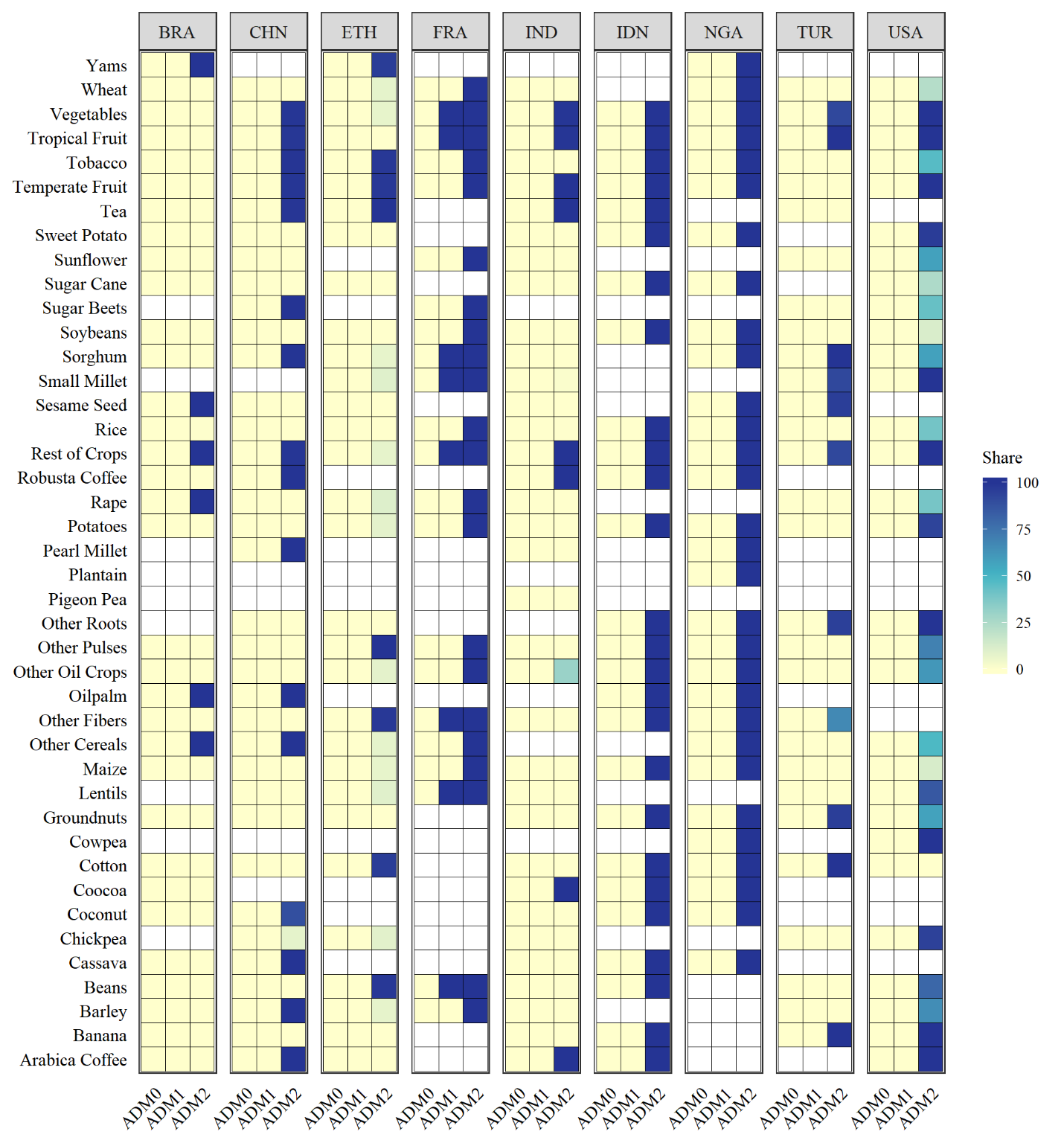
**

*Source:* Authors’ construction using data from You et al. (2017).

*Note:* BRA – Brazil; CHN – China; ETH – Ethiopia; FRA – France; IND – India; IDN – Indonesia; NGA – Nigeria; TUR – Turkey; USA – United States.

reported for those counties that produce cotton. For each of the other crops, the county-level coverage varies due to the United States non-disclosure policies, which ensure anonymity of farm-level data in counties where a limited number of farms produce a given crop. Indonesia and Nigeria have complete crop coverage at the ADM1-level, while in France data on eight crops and crop aggregates (beans, lentils, other fibers, rest-of-crops, small millet, sorghum, tropical fruit and vegetables) are reported only at the national-level in the sources available to the authors of the SPAM2005 estimates.

SPAM2005 disaggregated the reported crop statistics by production system using the share of area in each of four production systems (namely, irrigated, rainfed-high inputs, rainfed-low inputs and rainfed-subsistence). Irrigated production shares were derived by dividing the harvested area cultivated under irrigation, collected primarily from AQUASTAT (FAO 2012b), MIRCA (Portmann 2010) and from national statistics where available, by the total harvested area. The remaining area shares for each of the three rainfed production systems were determined on the basis of various “rules-of-thumb” differentiated by individual countries and crops. For example, fertilized areas were classified as either irrigated or rainfed-high systems, so if known, non-fertilized areas could be split between rainfed-low input and subsistence production systems. Production system shares varied significantly among the nine countries studied in this analysis. In France and China, a substantial share of maize was irrigated (48.7 and 41.3 percent respectively), while 14.1 percent was irrigated in the United States and approximately 1.0 percent in Nigeria, Brazil and Ethiopia. Three countries (China, France and the United States) had little to no rainfed-low input or subsistence maize production, while anywhere from 57.9 to 99.1 percent of maize production in Brazil, Ethiopia, India, Nigeria and Turkey (depending on the country and the location within each country) occurred under these two low-input systems.

Estimates of subnational physical area under production were derived from reported harvested area and indicators of seasonal production or multi-cropping. Limited data on cropping intensities existed for some countries and crops, but quite often cropping intensities were determined by consulting relevant grey literature or were based on the (informed) judgment of the SPAM2005 modelers. Cropping intensity values were generally 1.0 in temperate and cool climates, and for crops which had long growing periods, such as sugar cane or coconuts. Cropping intensities larger than 1.0 were common for irrigated crops, like vegetables, rice and maize. The highest cropping intensity for crops within the nine countries of interest was 2.5 for rice in the Hainan province of China, Taiwan and the Daerah Istimewa Yogyakarta region of Indonesia, as well as 2.5 for maize, cassava and vegetables in the Akwa Ibom state of Nigeria.

The biophysical constraints on crop production were modeled by interacting three pixelated datasets on cropland, irrigated land and agro-ecological suitability. SPAM2005 used the global, 30 arc-second resolution (approximately one kilometer at the equator) cropland map developed by the International Institute for Applied Systems Analysis (IIASA) and the International Food Policy Research Institute (IFPRI) which represents the median and maximum estimates of cropland circa 2005 (Fritz et al. 2015). These data were validated against high-resolution, remote-sensed data collected through two independent crowdsourcing campaigns and determined to have an accuracy of 82.4 percent globally. Within the countries of interest in this paper, most of the subnational cropland areas in the United States, China, France and Ethiopia were designated as being estimated with mid- to high-confidence levels; cropland areas in parts of India, Nigeria and Brazil were designated as being estimated with high-confidence, cropland areas elsewhere in the world were deemed estimated with low confidence, as, notably, was most of Indonesia and Turkey, two agriculturally important countries by global standards.

Pixelated estimates of irrigated areas were taken from the Global Map of Irrigation Areas (GMIA) version 5.0 which represents the amount of area equipped for irrigation circa 2005 at a 5 arc-minute resolution (Siebert et al. 2007). The estimated area equipped for irrigation (as a percentage of arable land) in the focus countries was 6.5 percent in Brazil, 55.5 percent in China, 2.3 percent in Ethiopia, 11.4 percent in France, 38.9 percent in India, 29.2 percent in Indonesia, 0.8 percent in Nigeria, 22.4 percent in Turkey and 17.3 percent in the United States.

Agro-ecological attributes are spatially sensitive and certain crops perform better under certain thermal, moisture, and soil conditions relative to others. IIASA and FAO (2012) developed the GAEZ (Global Agro-Ecological Zone) methodology to provide a standardized framework for assessing the spatially variable biophysical suitability of land for crop production worldwide. Using a set of agro ecological attributes, GAEZv3.0 produced a crop cum production-system specific index of land suitability at a 5 arc-minute resolution. These suitability indexes were used to designate the respective crop-specific suitability of the total land in crops used in forming the SPAM2005 estimates and the variants we assessed. Maps of the designated suitable area under irrigation, rainfed-high inputs and rainfed-low inputs are presented by Wood-Sichra et al. (2016). There is sizable variation among our nine focus countries in the area deemed suitable for crop production. Prior to forming the pixelated crop-specific estimates, the total cropland, irrigated area and suitable area in each pixel were re-calibrated according to the steps in Wood-Sichra et al. (2016, Appendix C) to satisfy the overall constraints on physical area set by the subnational statistics.

SPAM2005 included a measure of potential revenue in the model to take account of farmers’ decisions to plant one crop versus another. Potential revenue was modeled using crop-specific (but spatially invariant) prices derived from the FAOSTAT’s Gross Production Value (constant 2004–2006 International Dollars (I$)) (FAO 2012d) and a measure of accessibility based on the Global Rural and Urban Mapping Project, Version 1 estimates of rural population density (CIESIN et al. 2011; Balk et al. 2006) and potential yields as reported by GAEZv3.0 (IIASA and FAO 2012). Potential revenue was calculated on a pixelated basis for all crops and production systems.

## Processing

The estimates on potential revenue were combined with cropland and irrigated area to calculate a *prior* for physical area by crop and production system within each pixel. Using a cross-entropy allocation approach,^[[1]](#footnote-1)^ these priors were fed into a General Algebraic Modeling System (GAMS) based model to (iteratively) minimize the error between pre-allocated shares of physical area (i.e., the priors) and an allocated share of physical area in each pixel $i$ by crop $j$ and production system $l$, subject to several constraints. The model constraints specified the necessary relationships between the allocated physical area by pixel and cropland, suitable area, irrigated area and statistical physical area. If the model did not solve, a series of corrective measures were used including (i) adjusting the pre-processing parameters (i.e., the extent of cropland, irrigated land and suitable area), (ii) adjusting the entropy model constraints, and (iii) adjusting the data harmonization rules (e.g., applying different production shares to crop aggregates). The resulting estimates of physical area were converted to estimates of harvested area, production and yield.^[[2]](#footnote-2)^

## References

Balk, D. L., U. Deichmann, G. Yetman, F. Pozzi, S. I. Hay, and A. Nelson. 2006. "Determining Global Population Distribution: Methods, Applications and Data." *Advances in Parasitology* 62: 119-156.

CIESIN, IFPRI, the World Bank and CIAT. 2011. The Global Rural-Urban Mapping Project, Version 1 (GRUMPv1): Population Count Grid. Palisades, NY: NASA Socioeconomic Data and Applications Center (SEDAC). Available from URL: http://sedac.ciesin.columbia.edu/data/set/grump-v1-population-count [Accessed January 2015].

de Boer, P.T., D.P. Kroese, S. Mannor, and R.Y. Rubinstein. 2005. "A Tutorial on the Cross-Entropy Method." *Annals on Operations Research* 134: 19-67.

European Commison. 2008. "Nomenclature of Territorial Units for Statistics (NUTS) 2006 - Statistical Units - Data set." Eurostat. GISCO. Available from URL: http://ec.europa.eu/eurostat/web/gisco/geodata/reference-data/administrative-units-statistical-units/nuts#nuts06 [Accessed November 2017].

—. 2012. "Crops Products." Eurostat. Available from URL: http://ec.europa.eu/eurostat/data/database [Accessed May 2012].

FAO (Food and Agriculture Organization of the United Nations). 2012a. AGROMAPS Database Collection. Rome. Availabile from URL: http://kids.fao.org/agromaps/ [Accessed June 2012].

—. 2012b. AQUASTAT Database Collection. Rome. Availabile from URL: http://www.fao.org/nr/water/aquastat/sets/index.stm [Accessed June 2012].

—. 2012c. CountrySTAT Database Collection. Rome. Availabile from URL: http://countrystat.org/ [Accessed June 2012].

—. 2012d. FAOSTAT Database Collection. Rome. Availabile from URL: http://www.fao.org/faostat/en/#data [Accessed May 2012].

—. 2014. Global Administrative Unit Layers (GAUL). Available from URL: http://www.fao.org/geonetwork/srv/en/metadata.show?id=12691 [Accessed March 2014].

Fritz, S., L. See, I. McCallum, L. You, A. Bun, E. Moltchanova, M. Duerauer, F. Albrecht, C. Schill, C. Perger, P. Havlik, A. Mosnier, P. Thornton, U. Wood-Sichra, M. Herrero, I. Becker-Reshef, C. Justice, M. Hansen, P. Gong, S. Abdel Aziz, A. Cipriani, R. Cumani, G. Cecchi, G. Conchedda, S. Ferreira, A. Gomez, M. Haffani, F. Kayitakire, J. Malanding, R. Mueller, T. Newby, A. Nonguierma, A. Olusegun, S. Ortner, D.R. Rajak, J. Rocha, D. Schepaschenko, M. Schepaschenko, A. Terekhov, A. Tiangwa, C. Vancutsem, E. Vintrou, W. Wenbin, M. van der Velde, A. Dunwoody, F. Kraxner and M. Obersteiner. 2015. “Mapping Global Cropland and Field Size.” *Global Change Biology*, 21: 1980–1992. Available from URL: http://www.wur.nl/nl/Publicatie-details.htm?publicationId=publication-way-333433393832 [Accessed December 2016].

GADM. 2015. Database of Global Administrative Areas Version 2.8. Available from URL: http://www.gadm.org/ [Accessed November 2017].

IIASA and FAO. 2012. "Model Documentation." *Global Agro-ecological Zones (GAEZ v3.0).* Laxenburg, Austria and Rome, Italy: International Institute for Applied Systems Analysis and the Food and Agriculture Organization of the United Nations. Available from URL: http://www.fao.org/fileadmin/user_upload/gaez/docs/GAEZ_Model_Documentation.pdf [Accessed September 2015].

Portmann, F.T., S. Siebert, and P. Döll. 2010. "MIRCA2000-Global Monthly Irrigated and Rainfed Crop Areas Around the Year 2000: A New High Resolution Data Set for Agricultural and Hydrological Modeling." *Global Biogeochemical Cycles* 24: pp. 24.

Siebert, S., P. Döll, S. Feick, K. Frenken, and J. Hoogeveen. 2007. "Global Map of Irrigated Areas Version 4.0.1." Frankfurt, Germany and Rome, Italy: University of Frankfurt (Main) and the Food and Agriculture Organization of the United Nations. Available from URL: http://www.fao.org/nr/water/aquastat/irrigationmap/index10.stm [Accessed July 2012].

Wood-Sichra, U., A.B. Joglekar, and L. You. 2016. "Spatial Production Allocation Model (SPAM) 2005: Technical Documentation." *HarvestChoice Working Paper.* Washington, D.C.: International Food Policy Research Institute (IFPRI) and St. Paul: International Science and Technology Practice and Policy (InSTePP) Center, University of Minnesota.

You, L., U. Wood-Sichra, S. Fritz, Z. Guo, L. See, and J. Koo. 2017. Spatial Production Allocation Model (SPAM) 2005 version 3 release 1. *HarvestChoice Data Product*. Washington, D.C.: International Food Policy Research Institute (IFPRI) and St. Paul: International Science and Technology Practice and Policy (InSTePP) Center, University of Minnesota [Accessed May 2017].

1. de Boer et al. (2005) offer a good introduction to the cross-entropy method. [↑](#footnote-ref-1)
2. In our analysis, to remove outliers we set aside pixels with less than or equal to 0.1 hectares of total harvested area: the relevant 5 arc-minute pixels range from approximately 2,800 hectares to 8,500 hectares. [↑](#footnote-ref-2)
