# Supplementary material for: Pixelating crop production: Consequences of methodological choices
Source: PLoS One. 2019 Feb 19;14(2):e0212281. doi: 10.1371/journal.pone.0212281 (PMC6380596; doi:10.1371/journal.pone.0212281)
Supplement: S2 Appendix — (DOCX) [file pone.0212281.s002.docx]

# S2 Appendix

There are eight major methodological cum data choices examined in this analysis: the method used to spatially allocate areal data, the number of crops and crop aggregates included, the treatment of a “rest-of-crops” aggregate, the incorporation of a “crop suitability” layer, the inclusion of rudimentary economic elements, and the granularity of the administrative boundaries that spatially delineated the source statistics used to prime the allocation procedure.

## Allocation Method

There are two main types of spatial allocation models: (1) simple models that only use the cropland layer to inform spatial distributions of crops (Monfreda et al. 2008; Portmann et al. 2010) and (2) complex, optimization models that attempt to account for the localized, bio-economic decision environment of the farmer through the inclusion of several sources of geo-spatial information (Fischer et al. 2013; You et al. 2014; Wood-Sichra et al. 2016). The first method has computational advantages, but the second arguably captures more nuanced factors of influence. You and Wood (2006) compared the effectiveness of alternative spatial allocation models in explaining the variance in municipality crop areas (from a secondary database) in Brazil and found that the cross-entropy approach in SPAM2005 fared better than simpler methods.

In the first robustness test, the original estimates from SPAM2005’s cross-entropy optimization model are compared with those from a proportional allocation model similar to that used to derive the Monfreda et al. (2008) estimates of circa 2000 global harvested area and, subsequently, yield by crop. Monfreda et al. (2008) first collected tabulated statistics on harvested area and yield for the most disaggregated administrative unit (i.e., ADM0, ADM1 or ADM2) they could find, then downscaled the statistics on harvested area for *each crop* into pixels using just the share of *total cropland* within the respective administrative unit $k$ of interest:

(S2-1) $CropH_{ij}=CropLand_{i}\times\frac{CropH_{jk}}{\sum_{i\in k} Cropland_{i}}$

where $CropH_{ij}$ is the estimated harvested area in pixel $i$ for crop $j$, $CropH_{jk}$ is the total harvested area of crop $j$ in administrative unit $k$ and $CropLand_{i}$ is the total cropland area in each pixel $i$. Pixelated estimates of yield were subsequently allocated by assigning the respective administrative-level yield to each pixel with positive harvested area:

(S2-2) $\begin{matrix} CropY_{ij}=CropY_{jk} & if CropH_{ij}>0 \\ CropY_{ij}=0 & if CropH_{ij}=0 \end{matrix}$

where $CropY_{ij}$ is the estimated yield of crop $j$ in pixel $i$ and $CropY_{jk}$ is the statistical yield of crop $j$ in administrative unit $k$. Pixelated estimates of harvested area were then adjusted for multi-harvest potential. Details on the spatial allocation procedure used to calculate the pixilated production estimates used in our robustness test are presented in S3 Appendix.

To facilitate this robustness test, we aggregated the SPAM2005 estimates on crop-specific harvested area and production quantity to the SRU-level for each country, and then imputed the respective crop yield for each SRU. These SRU aggregates were then used to prime the spatial allocation procedure.

## Crop Choice

Certain locations will be more suitable (both biologically and economically) to particular crops. If a farmer is deciding between multiple crops. A decision to plant one crop over another will be dictated in part by local bio-economic factors. Thus, the more we know about the local variation in these bio-economic factors, the more accurate the pixelated production estimates are likely to be. SPAM2005 simultaneously allocates physical area to 41 crops and crop aggregates and one catchall (or residual) crop aggregate within a pixel conditioned on the bio-economic suitability of each crop to each pixel. We examine the effects of having less information on minor crops by re-running the model with only 33 crops and crop aggregates and a larger catchall crop aggregate. Table A contains a comparison of the crops that constituted the original SPAM2005 crop mix and those in the alternative crop mix included in our robustness assessment.

**Table A. Lookup table between original and alternative crops**

| # | Original Crop List | # | Alternative Crop List |  |  | # | Original Crop List | # | Alternative Crop List |
| --- | --- | --- | --- | --- | --- | --- | --- | --- | --- |
| 1 | wheat | 1 | wheat |  |  | 22 | coconut | 20 | coconut |
| 2 | rice | 2 | rice |  |  | 23 | oilpalm | 21 | oilpalm |
| 3 | maize | 3 | maize |  |  | 24 | sunflower | 22 | sunflower |
| 4 | barley | 4 | barley |  |  | 25 | rapeseed | 23 | rapeseed |
| 5 | pearl millet | 5 | pearl millet |  |  | 26 | sesameseed | 24 | sesameseed |
| 6 | small millet | 6 | small millet |  |  | 27 | other oil crops | 25 | other oil crops |
| 7 | sorghum | 7 | sorghum |  |  | 28 | sugarcane | 26 | sugarcane |
| 8 | other cereals | 34 | other crops |  |  | 29 | sugar beet | 27 | sugar beet |
| 9 | potato | 8 | potato |  |  | 30 | cotton | 28 | cotton |
| 10 | sweet potato | 9 | sweet potato |  |  | 31 | other fibre crops | 29 | other fibre crops |
| 11 | yams | 10 | yams |  |  | 32 | arabica coffee | 30 | arabica coffee |
| 12 | cassava | 11 | cassava |  |  | 33 | robusta coffee | 31 | robusta coffee |
| 13 | other roots | 34 | other crops |  |  | 34 | cocoa | 34 | other crops |
| 14 | bean | 12 | beans |  |  | 35 | tea | 34 | other crops |
| 15 | chickpea | 13 | chickpea |  |  | 36 | tobacco | 34 | other crops |
| 16 | cowpea | 14 | cowpea |  |  | 37 | banana | 32 | banana |
| 17 | pigeonpea | 15 | pigeonpea |  |  | 38 | plantain | 33 | plantain |
| 18 | lentil | 16 | lentil |  |  | 39 | tropical fruit | 34 | other crops |
| 19 | other pulses | 17 | other pulses |  |  | 40 | temperate fruit | 34 | other crops |
| 20 | soybean | 18 | soybean |  |  | 41 | vegetables | 34 | other crops |
| 21 | groundnut | 19 | groundnut |  |  | 42 | other crops | 34 | other crops |

*Source:* Wood-Sichra et al. (2016).

## Remainder Allocation

A “rest-of-crops” aggregate is a catchall for the minor crops not covered by the other 41 crops and crop aggregates (e.g., spices, tree nuts, other sugar crops, mate and rubber) in SPAM2005, but which are reported by FAO (2012d). In SPAM2005, data were collected for this rest-of-crops aggregate and modeled simultaneously with the other crops. However, in SPAM2000, the “rest-of-crops” aggregate was allocated after the model was run, to account for any residual or “unused” cropland. To examine potential differences between these two “rest-of-crops” allocation methods, we re-run the SPAM2005 data using the passive allocation method used to form the SPAM2000 estimates.

## Crop Suitability

The overall area deemed suitable for crop production is one of the major spatially delineating data layers in SPAM2005. Its importance is assessed by removing suitable area as a constraint in the allocation optimization model.^[[1]](#footnote-1)^ This constraint specifies that the sum of the physical area within each pixel allocated to each crop and production system cannot exceed the relevant crop-suitable area within each pixel. Removing this constraint may cause the model to allocate crops to unsuitable areas, which could overestimate the production and cropped area occurring in less suitable pixels.

## Economic Suitability

Economic suitability, as represented by potential revenue, is a function of market access, global crop prices and potential yields in SPAM2005. We test two rudimentary economic elements included in this revenue function: market access and crop prices. The proximity of a market to sell outputs or acquire inputs will influence the farmers’ management decisions (e.g., use of fertilizers, high-yielding seed varieties or machinery and timing of harvest). In SPAM2005, a measure of market access is derived from pixelated estimates of rural population density. For the robustness test, this variable is set to a constant across all pixels.

To measure the impact of crop prices within SPAM2005, the model is re-run without variation in crop prices (i.e., all prices are set to I$1.00/mt). Given the tradeoff between two crops, farmers will likely choose to plant the more profitable crop, *ceteris paribus*, so removing this layer may alter the total harvested area under a particular crop within a pixel. However, a global price layer may not accurately reflect the localized profitability (or more specifically revenue) trade-offs made by farmers at any given locale (where they are facing locally variable, not average global, prices).

## Administrative Level of Statistics

The final robustness scenario focuses on the aggregation of crop statistics. Here we assess the notion that pixilated estimates derived from cropping system models will be more accurate if more spatially disaggregated area crop statistics are used to prime the allocation procedure (e.g., more granular ADM2-level statistics are used rather than ADM1-level data). However, data at these subnational, albeit still course resolutions, can be difficult to come by. For example, agricultural household surveys may only be spatially representative at either an ADM0- or ADM1-level, but not at an ADM2-level, although in some cases, a survey will include a sufficiently large number of spatial dispersed respondents to enable representative ADM2-level estimates to be formed (e.g., Tanzania’s 2007 Agricultural Sample Census, NBS 2011).

The SPAM2005 model is run on a country-by-country basis and is mostly primed with ADM1-level data^[[2]](#footnote-2)^, and, in some instances ADM2-level data. Lacking crop information at these finer spatial scales pixelated estimates for some countries are formed using only ADM0-level (i.e., country-level) data to prime the model. Priming the model with only ADM0-level data means that the resulting pixilated estimates are formed in a way that is entirely unconstrained by any subnational geographic boundaries. In these instances there is a higher likelihood of spatially misallocating crops, which is likely to be magnified when the relevant geopolitical units are large. Moreover, in some instances—specifically China, India and Nigeria—the maximum entropy allocation method was unable to solve the model when it was primed with country-level aggregates.

## References

FAO (Food and Agriculture Organization of the United Nations). 2012. FAOSTAT Database Collection. Rome. Availabile from URL: http://www.fao.org/faostat/en/#data [Accessed May 2012].

Fischer, G., F.O. Nachtergaele, S. Prieler, E. Teixeira, G. Tóth, H. van Velthuizen, L. Verelst, and D. Wiberg. 2013. Global Agro-Ecological Zones (GAEZ) Version 3.0. Available from URL: http://www.fao.org/nr/gaez/en/ [Accessed October 2014].

Monfreda, C., N. Ramankutty, and J.A. Foley. 2008. "Geographic Distribution of Crop Areas, Yields, Physiological Types, and Net Primary Production in the Year 2000." *Global Biogeochemical Cycles* 22: pp. 19.

NBS. 2011. "Agricultural Sample Census Survey 2007/2008 version 1.0." National Bureau of Statistics. Available from URL: www.nbs.go.tz [Accessed June 2014].

Portmann, F.T., S. Siebert, and P. Döll. 2010. "MIRCA2000-Global Monthly Irrigated and Rainfed Crop Areas Around the Year 2000: A New High Resolution Data Set for Agricultural and Hydrological Modeling." *Global Biogeochemical Cycles* 24: pp. 24.

Wood-Sichra, U., A.B. Joglekar, and L. You. 2016. "Spatial Production Allocation Model (SPAM) 2005: Technical Documentation." *HarvestChoice Working Paper.* Washington, D.C.: International Food Policy Research Institute (IFPRI) and St. Paul: International Science and Technology Practice and Policy (InSTePP) Center, University of Minnesota.

You, L., and S. Wood. 2006. "An Entropy Approach to Spatial Disaggregation of Agricultural Production." *Agricultural Systems* 90: 329-347.

You, L., S. Wood, U. Wood-Sichra, and W. Wu. 2014. "Generating Global Crop Distribution Maps: From Census to Grid." *Agricultural Systems* 127: 53-60.

1. Suitable area was still used to adjust cropland and irrigated areas, as described in Wood-Sichra et al. (2016, Appendix B). [↑](#footnote-ref-1)
2. Access to subnational data is improving—81 percent of the year 2000 global harvested area data used in Monfreda et al. (2008) came from subnational sources, while 93.4 percent of the year 2005 global harvested area data used in SPAM2005 came from ADM1 sources and 54.6 percent from ADM2 sources. The few countries lacking any subnational administratively delineated statistics in SPAM2005 are typically geographically small or politically unstable countries. Even though countries are increasingly collecting subnational production data, not all crops are included because some crops are not as economically significant as others or reporting such data could reveal confidential information. All nine of the countries included in this analysis had most crop production statistics available at an ADM1-level data (for eight crops in France, data was only available at a national level). Six countries in this analysis had at least some crop statistics available at an ADM2-level (see Fig A in S1 Appendix). [↑](#footnote-ref-2)
