# Supplementary material for: Pixelating crop production: Consequences of methodological choices
Source: PLoS One. 2019 Feb 19;14(2):e0212281. doi: 10.1371/journal.pone.0212281 (PMC6380596; doi:10.1371/journal.pone.0212281)
Supplement: S3 Appendix — (DOCX) [file pone.0212281.s003.docx]

# S3 Appendix

The alternative allocation method we evaluate is equivalent to the simple proportional allocation method used by used Monfreda et al. (2008) to form their M3-Crop estimates. To implement this robustness test, the original SPAM2005 estimates of pixilated harvested area ($H$) and yield $(Y$), differentiated by production systems (i.e., irrigated, rainfed-high inputs, rainfed-low inputs and rainfed-subsistence), were first re-aggregated to national (ADM0) and SRU (predominately ADM2) administrative boundaries.^[[1]](#footnote-1)^ These re-aggregated statistics were then used to prime the allocation procedure using a simple proportional allocation method.

To implement this method, the SRU estimates of harvested area for *each crop* were first “downscaled” into pixels using the share of *total cropland* within each of the respective SRUs, $k_{SRU}$ such that:

(S3-1) $CropH_{ij}=CropH_{jk_{SRU}}\times\frac{CropLand_{i}}{\sum_{i\in k_{SRU}} CropLand_{i}},$

where $CropH_{ij}$ is the estimated harvested area in pixel $i$ from crop $j$, $CropH_{jk_{SRU}}$ is the total harvested area of crop $j$ in SRU $k_{SRU}$ and $CropLand_{i}$ is the total cropland area in each pixel $i$. The data on the pixelated value of cropland used in SPAM2005 were sourced from Fritz et al. (2015). Cropland was adjusted using irrigated area and suitable area as described in Wood-Sichra et al. (2016). To create a gridded map of yield, SRU-level statistics on yield were uniformly distributed to pixels with positive harvested area such that:

(S3-2) $\begin{matrix} CropY_{ij}=CropY_{jk_{SRU}} & if CropH_{ij}>0 \\ CropY_{ij}=0 & if CropH_{ij}=0 \end{matrix},$

where $CropY_{ij}$ is the estimated yield of crop $j$ in pixel $i$ and $CropY_{jk_{SRU}}$ is the statistical yield of crop $j$ in SRU $k_{SRU}$.

Following Monfreda et al. (2008), we corrected for possible instances of harvested area that exceed the multiple cropping potential of the land by adjusting the gridded harvested area estimates with *harvest ratios* that were greater than the relevant cropping intensities used within SPAM2005. Harvest ratios were calculated as the ratio of total harvested area (across all crops $j$) and cropland within pixel $i$

(S3-3) $HarvRatio_{i}=\frac{\sum_{j} CropH_{ij}}{CropLand_{i}}, \forall j.$

SPAM2005 compiled evidence on the cropping intensities (by crop) at both an ADM0- and ADM-level for irrigated ($CIIRR_{jk}$), rainfed-high ($CIRFH_{jk}$) and rainfed-low ($CIRFL_{jk}$) production systems (subsistence systems were assumed to have the same cropping intensities as rainfed-low systems). The rainfed cropping intensity ($CIRF_{jk}$) by crop $j$ and administrative unit $k\in k_{0},k_{1}$ was calculated as a weighted share of the high and low input cropping intensities,

(S3-4) $CIRF_{jk}=SHRFH_{jk}\times CIRFH_{jk}+\left( 1-\left( SHIRR_{jk}+SHRFH_{jk} \right) \right)\times CIRFL_{jk}, \forall j\forall k\in k_{0},k_{1},$

where the weights $SHIRR_{jk}$ and $SHRFH_{jk}$ are the share of harvested area by crop $j$ and administrative unit $k$ for irrigated and rainfed-high systems, respectively. To create a single cropping intensity, only the ADM-level cropping intensities were used. However, if an ADM1-level cropping intensity was equal to zero, its value was replaced with its respective ADM0-level cropping intensity

(S3-5a) $\begin{matrix} CIRR_{jk_{1}}=CIRR_{jk_{1}} & if CIRR_{jk_{1}}>0 \\ CIRR_{jk_{1}}=CIRR_{jk_{0}} & Otherwise \end{matrix},$

(S3-5b) $\begin{matrix} CIRF_{jk_{1}}=CIRF_{jk_{1}} & if CIRF_{jk_{1}}>0 \\ CIRF_{jk_{1}}=CIRF_{jk_{0}} & Otherwise \end{matrix}.$

Cropping intensities were downscaled to a pixel-level by uniformly distributing both irrigated and rainfed cropping intensities to pixels based on crop $j$ and administrative unit $k$

(S3-6a) $CIIRR_{ij}=CIIRR_{jk_{1}},$

(S3-6b) $CIRF_{ij}=CIRF_{jk_{1}}.$

These cropping intensities were then averaged over all crops using the pixel-level harvested area as weights

(S3-7a) $CIRR_{i}=CIRR_{ij}\times\frac{CropH_{ij}}{\sum_{j} CropH_{ij}},$

(S3-7b) $CIRF_{i}=CIRF_{i}\times\frac{CropH_{ij}}{\sum_{j} CropH_{ij}}.$

Finally, the pixel-level irrigated and rainfed cropping intensities were averaged by pixel using area-weighted shares

(S3-8) $CropIntensity_{i}=SHIRR_{i}\times CIRR_{i}+\left( 1-SHIRR_{i} \right)\times CIRF_{i}.$

where $SHIRR_{i}$ is equal to the ratio of irrigated area to cropland in each pixel. The data on the pixelated value of irrigated area used in SPAM2005 were sourced from Siebert et al. (2007) and adjusted using cropland as described in Wood-Sichra et al. (2016).

Thus, pixels with a harvest ratio that exceed their cropping intensity were scaled downward to reflect the relevant cropping intensity^[[2]](#footnote-2)^

(S3-9) $\begin{matrix} AdjCro{pH}_{ij}=CropH_{ij}\times\frac{CropIntensity_{i}}{HarvRatio_{i}} & if HarvRatio_{i}>CropIntensity_{i} \\ AdjCro{pH}_{ij}=CropH_{ij} & Otherwise \end{matrix}$

Production quantity was subsequently calculated as

(S3-10) $CropP_{ij}=AdjCropH_{ij}\times CropY_{ij}.$

Since the associated values of yield with adjusted pixels were left uncorrected, there was an overall decrease in the total harvested area and production quantity across all crops at a national-level associated with adjusted pixels.

## References

Monfreda, C., N. Ramankutty, and J.A. Foley. 2008. "Geographic Distribution of Crop Areas, Yields, Physiological Types, and Net Primary Production in the Year 2000." *Global Biogeochemical Cycles* 22: pp. 19.

Siebert, S., P. Döll, S. Feick, K. Frenken, and J. Hoogeveen. 2007. "Global Map of Irrigated Areas Version 4.0.1." Frankfurt, Germany and Rome, Italy: University of Frankfurt (Main) and the Food and Agriculture Organization of the United Nations. Available from URL: http://www.fao.org/nr/water/aquastat/irrigationmap/index10.stm [Accessed July 2012].

Wood-Sichra, U., A.B. Joglekar, and L. You. 2016. "Spatial Production Allocation Model (SPAM) 2005: Technical Documentation." *HarvestChoice Working Paper.* Washington, D.C.: International Food Policy Research Institute (IFPRI) and St. Paul: International Science and Technology Practice and Policy (InSTePP) Center, University of Minnesota.

1. Aggregate harvested area was calculated by summing across all pixels (under all production systems) within each relevant administrative unit. Aggregate yield was calculated by summing area-weighted yields across all pixels (under all production systems) within each relevant administrative unit. [↑](#footnote-ref-1)
2. The correction only affected a minor number of pixels: zero pixels in France, Indonesia, Nigeria and Turkey, 0.7 percent of pixels in Brazil and the United States, 0.8 percent in Ethiopia, 1.3 percent in China and 2.2 percent in India. [↑](#footnote-ref-2)
