# Supplementary material for: Pixelating crop production: Consequences of methodological choices
Source: PLoS One. 2019 Feb 19;14(2):e0212281. doi: 10.1371/journal.pone.0212281 (PMC6380596; doi:10.1371/journal.pone.0212281)
Supplement: S4 Appendix — (DOCX) [file pone.0212281.s004.docx]

# S4 Appendix

Table A reports the regional-level (ADM1) maize presence data for the each of the nine countries of interest. Only the top ten regions of maize production (according to quantity) are reported for each country. Similar to Table 2 in the main text, Column 2 in Table A reports the share of total cropland pixels with positive maize production under the reported estimates. Columns 3-10 report the share of total crop pixels with positive maize production under each of the methodological cum data scenarios analyzed. For most countries, there are a few regions with marked differences in the estimated share of pixels growing maize among the scenarios we analyzed. For instance, the original SPAM2005 share of cropland pixels growing maize in Para, Brazil is 39.5 percent, while the Monfreda et al. (2008) allocation method puts the share at 93 percent. Some of the robustness tests (e.g., maize production in Mato Grosso do Sul, Brazil based on ADM0-level data), results in an implausible spatial allocation (in this instance indicate that 147.5 percent of the cropland pixels are planted to maize, compared with 67.9 percent under the published data). In the case of Ethiopia and Turkey, some of the robustness tests resulted in spatial allocations that varied markedly from the original SPAM2005 estimates. In countries like France, Nigeria and the United States, there are little to no differences between the modelled presence or absence of maize production by pixels for each of the scenarios examined.

## References

Fritz, S., L. See, I. McCallum, L. You, A. Bun, E. Moltchanova, M. Duerauer, F. Albrecht, C. Schill, C. Perger, P. Havlik, A. Mosnier, P. Thornton, U. Wood-Sichra, M. Herrero, I. Becker-Reshef, C. Justice, M. Hansen, P. Gong, S. Abdel Aziz, A. Cipriani, R. Cumani, G. Cecchi, G. Conchedda, S. Ferreira, A. Gomez, M. Haffani, F. Kayitakire, J. Malanding, R. Mueller, T. Newby, A. Nonguierma, A. Olusegun, S. Ortner, D.R. Rajak, J. Rocha, D. Schepaschenko, M. Schepaschenko, A. Terekhov, A. Tiangwa, C. Vancutsem, E. Vintrou, W. Wenbin, M. van der Velde, A. Dunwoody, F. Kraxner and M. Obersteiner. 2015. “Mapping Global Cropland and Field Size.” *Global Change Biology*, 21: 1980–1992. Available from URL: http://www.wur.nl/nl/Publicatie-details.htm?publicationId=publication-way-333433393832 [Accessed December 2016].

Monfreda, C., N. Ramankutty, and J.A. Foley. 2008. "Geographic Distribution of Crop Areas, Yields, Physiological Types, and Net Primary Production in the Year 2000." *Global Biogeochemical Cycles* 22: pp. 19.

Wood-Sichra, U., A.B. Joglekar, and L. You. 2016. "Spatial Production Allocation Model (SPAM) 2005: Technical Documentation." *HarvestChoice Working Paper.* Washington, D.C.: International Food Policy Research Institute (IFPRI) and St. Paul: International Science and Technology Practice and Policy (InSTePP) Center, University of Minnesota.

You, L., U. Wood-Sichra, S. Fritz, Z. Guo, L. See, and J. Koo. 2017. Spatial Production Allocation Model (SPAM) 2005 version 3 release 1. *HarvestChoice Data Product*. Washington, D.C.: International Food Policy Research Institute (IFPRI) and St. Paul: International Science and Technology Practice and Policy (InSTePP) Center, University of Minnesota [Accessed May 2017].

**Table A: Comparison of non-zero maize production pixels between original and robustness test estimates at ADM1-level**

|  |  |  |  |  |  | Robustness Tests | | | | | | | | | |
| --- | --- | --- | --- | --- | --- | --- | --- | --- | --- | --- | --- | --- | --- | --- | --- |
|  |  |  |  |  |  |  |  |  |  |  | Economic Suitability | |  | Underlying Statistics | |
|  |  | (1) |  | (2) |  | (3) | (4) | (5) | (6) |  | (7) | (8) |  | (9) | (10) |
| Region | | Cropland Pixels |  | Original |  | Allocation Method | Crop Choice | Remainder Allocation | Crop Suitability |  | Market Access | Crop Price |  | ADM0 Only | ADM1 Only |
|  |  | (count) |  | (percent) | | | | | | | | | | | |
| Brazil | |  |  |  | | | | | | | | | | | |
|  | Bahia | 6,313 |  | 86.0 |  | 92.5 | 87.3 | 85.7 | 88.2 |  | 86.1 | 85.2 |  | 95.5 | 88.4 |
|  | Goias | 4,084 |  | 85.8 |  | 98.9 | 84.3 | 86.3 | 86.3 |  | 85.9 | 86.0 |  | 99.3 | 77.0 |
|  | Mato Grosso | 8,891 |  | 93.5 |  | 97.8 | 94.1 | 94.3 | 94.5 |  | 94.2 | 94.2 |  | 99.4 | 94.3 |
|  | Mato Grosso do Sul | 2,791 |  | 67.9 |  | 96.0 | 68.1 | 68.0 | 78.0 |  | 68.0 | 68.0 |  | 147.5 | 73.7 |
|  | Minas Gerais | 6,909 |  | 92.3 |  | 98.0 | 92.5 | 92.2 | 93.3 |  | 92.3 | 92.1 |  | 102.9 | 82.4 |
|  | Para | 6,830 |  | 39.5 |  | 93.0 | 38.3 | 39.5 | 39.6 |  | 39.5 | 39.5 |  | 98.0 | 28.1 |
|  | Parana | 2,444 |  | 86.7 |  | 98.3 | 86.8 | 87.1 | 89.0 |  | 86.9 | 86.7 |  | 102.5 | 74.5 |
|  | Rio Grande do Sul | 3,192 |  | 85.7 |  | 96.1 | 84.8 | 85.8 | 91.2 |  | 85.7 | 85.7 |  | 112.4 | 79.7 |
|  | Santa Catarina | 1,174 |  | 80.5 |  | 95.6 | 80.2 | 80.5 | 83.6 |  | 80.2 | 80.4 |  | 103.8 | 61.0 |
|  | Sao Paulo | 2,977 |  | 94.4 |  | 96.4 | 94.4 | 94.6 | 93.9 |  | 94.3 | 94.2 |  | 102.8 | 92.8 |
|  |  |  |  |  |  |  |  |  |  |  |  |  |  |  |  |
| China | |  |  |  |  |  |  |  |  |  |  |  |  |  |  |
|  | Hebei | 2,723 |  | 85.5 |  | 86.2 | 80.2 | 79.9 | 85.4 |  | 85.0 | 85.6 |  |  | 86.9 |
|  | Heilongjiang | 4,831 |  | 80.5 |  | 87.5 | 80.9 | 78.0 | 83.2 |  | 80.1 | 80.5 |  |  | 82.1 |
|  | Henan | 2,185 |  | 87.0 |  | 91.0 | 91.7 | 86.5 | 90.1 |  | 87.3 | 87.0 |  |  | 85.8 |
|  | Inner Mongolia | 6,401 |  | 57.4 |  | 59.3 | 57.7 | 57.5 | 59.2 |  | 57.2 | 57.4 |  |  | 45.7 |
|  | Jilin | 2,474 |  | 88.5 |  | 96.4 | 95.1 | 90.1 | 90.1 |  | 87.6 | 88.6 |  |  | 87.9 |
|  | Liaoning | 2,195 |  | 84.6 |  | 86.7 | 83.8 | 84.7 | 83.9 |  | 83.6 | 84.2 |  |  | 76.4 |
|  | Shaanxi | 2,627 |  | 87.2 |  | 96.5 | 96.3 | 87.3 | 89.8 |  | 86.8 | 87.9 |  |  | 92.1 |
|  | Shandong | 2,319 |  | 92.8 |  | 94.1 | 91.4 | 92.9 | 92.8 |  | 92.8 | 92.8 |  |  | 94.1 |
|  | Shanxi | 2,025 |  | 81.4 |  | 83.6 | 81.5 | 81.4 | 84.1 |  | 81.0 | 81.3 |  |  | 82.4 |
|  | Sichuan | 3,451 |  | 76.3 |  | 75.3 | 75.6 | 76.4 | 77.3 |  | 75.4 | 76.3 |  |  | 51.2 |
|  |  |  |  |  |  |  |  |  |  |  |  |  |  |  |  |
| Ethiopia | |  |  |  |  |  |  |  |  |  |  |  |  |  |  |
|  | Afar | 65 |  | 100.0 |  | 92.3 | 98.5 | 100.0 | 100.0 |  | 100.0 | 100.0 |  | 96.9 | 104.6 |
|  | Amhara | 1,361 |  | 60.3 |  | 82.1 | 63.3 | 57.6 | 81.9 |  | 60.3 | 60.4 |  | 67.2 | 67.2 |
|  | Benishangul-Gumuz | 264 |  | 98.9 |  | 96.2 | 98.9 | 98.9 | 98.9 |  | 98.9 | 98.9 |  | 8.3 | 98.5 |
|  | Dire Dawa | 11 |  | 100.0 |  | 100.0 | 100.0 | 100.0 | 100.0 |  | 100.0 | 100.0 |  | 81.8 | 100.0 |
|  | Gambella | 59 |  | 71.2 |  | 67.8 | 71.2 | 71.2 | 71.2 |  | 71.2 | 71.2 |  | 11.9 | 71.2 |
|  | Harari | 3 |  | 100.0 |  | 100.0 | 100.0 | 100.0 | 100.0 |  | 100.0 | 100.0 |  | 100.0 | 100.0 |
|  | Oromia | 1,686 |  | 69.6 |  | 84.0 | 75.5 | 70.3 | 79.8 |  | 69.6 | 69.8 |  | 67.0 | 66.8 |
|  | SNNP | 590 |  | 73.9 |  | 93.2 | 53.4 | 73.7 | 81.2 |  | 73.7 | 73.6 |  | 75.8 | 50.8 |
|  | Somali | 168 |  | 47.0 |  | 53.0 | 47.0 | 47.6 | 54.2 |  | 47.6 | 47.0 |  | 51.8 | 82.1 |
|  | Tigray | 510 |  | 74.5 |  | 82.5 | 72.9 | 74.5 | 76.1 |  | 74.5 | 74.5 |  | 77.6 | 68.2 |
|  |  |  |  |  |  |  |  |  |  |  |  |  |  |  |  |
| France | |  |  |  |  |  |  |  |  |  |  |  |  |  |  |
|  | Alsace | 129 |  | 86.8 |  | 86.8 | 81.4 | 81.4 | 81.4 |  | 81.4 | 81.4 |  | 81.4 | 81.4 |
|  | Aquitaine | 624 |  | 101.8 |  | 99.0 | 98.9 | 98.9 | 98.9 |  | 98.9 | 98.9 |  | 98.9 | 98.9 |
|  | Bourgogne | 480 |  | 109.0 |  | 99.2 | 96.5 | 96.5 | 96.5 |  | 96.5 | 96.5 |  | 96.5 | 96.5 |
|  | Bretagne | 504 |  | 54.0 |  | 53.6 | 53.2 | 53.2 | 53.2 |  | 53.2 | 53.0 |  | 53.2 | 53.2 |
|  | Centre | 682 |  | 99.3 |  | 99.6 | 99.6 | 99.3 | 99.3 |  | 99.3 | 99.3 |  | 99.3 | 99.3 |
|  | Champagne-Ardenne | 361 |  | 114.4 |  | 96.4 | 112.7 | 114.7 | 114.7 |  | 114.7 | 114.7 |  | 105.3 | 114.7 |
|  | Midi-Pyrenees | 632 |  | 98.4 |  | 95.1 | 94.9 | 94.9 | 94.9 |  | 94.9 | 94.9 |  | 94.9 | 94.9 |
|  | Pays-de-la-Loire | 561 |  | 96.6 |  | 96.1 | 95.7 | 95.5 | 95.5 |  | 95.5 | 95.5 |  | 95.5 | 95.5 |
|  | Poitou-Charentes | 450 |  | 97.1 |  | 97.1 | 96.4 | 96.4 | 96.4 |  | 96.4 | 96.4 |  | 96.4 | 96.4 |
|  | Rhone-Alpes | 521 |  | 85.2 |  | 82.1 | 82.3 | 82.3 | 82.3 |  | 82.3 | 82.3 |  | 82.3 | 82.3 |
|  |  |  |  |  |  |  |  |  |  |  |  |  |  |  |  |
| Indian | |  |  |  |  |  |  |  |  |  |  |  |  |  |  |
|  | Andhra Pradesh | 3,414 |  | 83.1 |  | 83.8 | 82.6 | 83.0 | 83.5 |  | 83.0 | 83.1 |  |  |  |
|  | Bihar | 1,220 |  | 98.5 |  | 99.3 | 99.0 | 99.3 | 98.6 |  | 97.9 | 98.5 |  |  | 99.0 |
|  | Himachal Pradesh | 676 |  | 54.9 |  | 70.7 | 53.1 | 54.9 | 69.8 |  | 54.7 | 54.9 |  |  | 48.5 |
|  | Karnataka | 2,337 |  | 73.5 |  | 76.6 | 73.6 | 73.6 | 77.1 |  | 73.1 | 73.5 |  |  |  |
|  | Madhya Pradesh | 3,865 |  | 96.0 |  | 96.0 | 96.1 | 96.0 | 96.3 |  | 94.7 | 96.1 |  |  | 98.3 |
|  | Maharashtra | 3,845 |  | 82.3 |  | 83.3 | 82.2 | 82.3 | 81.1 |  | 83.0 | 82.3 |  |  |  |
|  | Punjab | 676 |  | 55.9 |  | 55.8 | 55.9 | 55.9 | 55.9 |  | 55.9 | 55.9 |  |  | 100.1 |
|  | Rajasthan | 4,150 |  | 44.4 |  | 53.9 | 44.5 | 48.9 | 52.1 |  | 44.4 | 48.0 |  |  | 87.5 |
|  | Tamil Nadu | 1,605 |  | 69.6 |  | 73.1 | 70.5 | 69.8 | 70.1 |  | 69.6 | 69.7 |  |  |  |
|  | Uttar Pradesh | 3,107 |  | 90.1 |  | 90.2 | 90.1 | 90.1 | 90.1 |  | 90.1 | 90.1 |  |  |  |
|  |  |  |  |  |  |  |  |  |  |  |  |  |  |  |  |
| Indonesia | |  |  |  |  |  |  |  |  |  |  |  |  |  |  |
|  | Daerah Istimewa Yogyakarta | 42 |  | 97.6 |  | 97.6 | 97.6 | 97.6 | 97.6 |  | 95.2 | 97.6 |  | 83.3 | 97.6 |
|  | Gorontalo | 167 |  | 97.6 |  | 97.0 | 97.6 | 97.6 | 97.6 |  | 97.6 | 97.6 |  | 80.2 | 97.6 |
|  | Jawa Barat | 474 |  | 89.0 |  | 99.6 | 89.0 | 89.0 | 98.3 |  | 88.8 | 89.0 |  | 91.1 | 89.0 |
|  | Jawa Tengah | 447 |  | 88.6 |  | 98.9 | 87.7 | 88.6 | 97.3 |  | 88.4 | 88.6 |  | 90.2 | 88.6 |
|  | Jawa Timur | 693 |  | 79.8 |  | 97.7 | 79.2 | 79.8 | 96.8 |  | 79.5 | 79.8 |  | 81.4 | 79.8 |
|  | Lampung | 437 |  | 87.0 |  | 95.2 | 87.9 | 87.0 | 92.9 |  | 86.0 | 87.0 |  | 89.0 | 87.0 |
|  | Nusatenggara Timur | 764 |  | 73.4 |  | 84.2 | 73.4 | 55.6 | 73.4 |  | 73.0 | 73.4 |  | 71.6 | 55.6 |
|  | Sulawesi Selatan | 611 |  | 55.5 |  | 88.4 | 55.8 | 55.5 | 55.8 |  | 55.5 | 55.5 |  | 84.5 | 55.5 |
|  | Sulawesi Utara | 262 |  | 98.9 |  | 96.6 | 99.2 | 98.9 | 98.9 |  | 98.9 | 98.9 |  | 66.0 | 98.9 |
|  | Sumatera Utara | 931 |  | 83.4 |  | 95.5 | 83.6 | 90.3 | 88.5 |  | 81.4 | 83.4 |  | 90.3 | 90.3 |
|  |  |  |  |  |  |  |  |  |  |  |  |  |  |  |  |
| Nigeria | |  |  |  |  |  |  |  |  |  |  |  |  |  |  |
|  | Adamawa | 356 |  | 88.2 |  | 88.2 | 88.2 | 88.2 | 88.2 |  | 88.2 | 88.2 |  |  | 88.2 |
|  | Borno | 738 |  | 83.5 |  | 84.4 | 83.5 | 83.5 | 84.4 |  | 83.5 | 83.5 |  |  | 83.5 |
|  | Gombe | 210 |  | 97.6 |  | 97.6 | 97.6 | 96.2 | 97.6 |  | 97.6 | 97.6 |  |  | 97.6 |
|  | Kaduna | 527 |  | 100.0 |  | 100.0 | 100.0 | 100.0 | 100.0 |  | 100.0 | 100.0 |  |  | 100.0 |
|  | Kogi | 336 |  | 99.7 |  | 100.0 | 100.0 | 99.7 | 99.7 |  | 99.7 | 99.7 |  |  | 99.7 |
|  | Niger | 819 |  | 81.1 |  | 81.1 | 81.1 | 72.3 | 81.1 |  | 81.1 | 81.1 |  |  | 81.1 |
|  | Ondo | 184 |  | 74.5 |  | 82.6 | 83.2 | 74.5 | 74.5 |  | 74.5 | 74.5 |  |  | 74.5 |
|  | Oyo | 326 |  | 99.7 |  | 99.7 | 99.7 | 99.7 | 99.7 |  | 99.4 | 99.7 |  |  | 99.7 |
|  | Plateau | 324 |  | 96.0 |  | 96.0 | 96.0 | 96.0 | 96.0 |  | 96.0 | 96.0 |  |  | 96.0 |
|  | Taraba | 657 |  | 86.6 |  | 97.3 | 97.3 | 87.1 | 91.9 |  | 78.1 | 86.6 |  |  | 86.6 |
|  |  |  |  |  |  |  |  |  |  |  |  |  |  |  |  |
| Turkey | |  |  |  |  |  |  |  |  |  |  |  |  |  |  |
|  | Akdeniz | 1,382 |  | 69.7 |  | 99.6 | 69.9 | 69.7 | 99.4 |  | 69.7 | 69.7 |  | 69.5 | 69.8 |
|  | Bati Anadolu | 1,095 |  | 79.0 |  | 97.5 | 49.0 | 79.1 | 95.1 |  | 78.9 | 79.0 |  | 81.8 | 81.4 |
|  | Bati Karadeniz | 1,185 |  | 68.9 |  | 92.3 | 68.9 | 68.9 | 80.5 |  | 68.9 | 68.9 |  | 80.9 | 68.9 |
|  | Bati Marmara | 722 |  | 90.6 |  | 93.8 | 90.6 | 81.6 | 93.9 |  | 90.6 | 90.6 |  | 90.6 | 90.6 |
|  | Dogu Karadeniz | 578 |  | 52.8 |  | 100.0 | 52.8 | 43.1 | 100.9 |  | 52.8 | 52.8 |  | 52.8 | 52.8 |
|  | Dogu Marmara | 773 |  | 81.9 |  | 95.9 | 81.9 | 71.7 | 88.6 |  | 81.9 | 81.9 |  | 89.3 | 81.9 |
|  | Ege | 1,392 |  | 84.8 |  | 99.6 | 84.8 | 84.8 | 100.0 |  | 84.8 | 84.8 |  | 85.1 | 84.8 |
|  | Güneydogu Anadolu | 1,137 |  | 100.0 |  | 100.0 | 100.0 | 100.0 | 100.0 |  | 100.0 | 100.0 |  | 96.5 | 100.0 |
|  | Orta Anadolu | 1,350 |  | 63.9 |  | 66.7 | 65.1 | 64.0 | 63.9 |  | 63.8 | 64.4 |  | 59.0 | 64.0 |
|  | Ortadogu Anadolu | 1,194 |  | 57.9 |  | 73.6 | 57.2 | 57.9 | 69.0 |  | 58.0 | 57.6 |  | 64.8 | 60.9 |
|  |  |  |  |  |  |  |  |  |  |  |  |  |  |  |  |
| United States | |  |  |  |  |  |  |  |  |  |  |  |  |  |  |
|  | Illinois | 2,114 |  | 100.1 |  | 99.8 | 100.1 | 100.1 | 100.1 |  | 100.2 | 100.1 |  | 101.8 | 99.4 |
|  | Indiana | 1,291 |  | 99.5 |  | 98.2 | 99.5 | 99.5 | 99.5 |  | 99.5 | 99.5 |  | 98.5 | 98.5 |
|  | Iowa | 2,253 |  | 100.0 |  | 100.0 | 100.0 | 100.0 | 100.0 |  | 100.1 | 100.0 |  | 100.8 | 100.0 |
|  | Kansas | 3,080 |  | 100.1 |  | 98.7 | 100.1 | 100.1 | 100.2 |  | 100.1 | 100.1 |  | 97.9 | 99.1 |
|  | Minnesota | 2,521 |  | 91.2 |  | 90.8 | 91.2 | 91.2 | 92.7 |  | 91.2 | 91.2 |  | 101.1 | 100.0 |
|  | Missouri | 1,858 |  | 96.1 |  | 92.0 | 96.1 | 96.1 | 96.1 |  | 96.1 | 96.1 |  | 102.3 | 95.7 |
|  | Nebraska | 2,733 |  | 99.9 |  | 98.7 | 99.9 | 99.9 | 100.0 |  | 99.9 | 99.9 |  | 98.7 | 98.7 |
|  | Ohio | 1,295 |  | 98.0 |  | 96.6 | 98.0 | 98.0 | 98.0 |  | 98.0 | 98.0 |  | 97.0 | 92.5 |
|  | South Dakota | 2,334 |  | 105.3 |  | 98.0 | 105.3 | 105.3 | 105.9 |  | 105.3 | 105.3 |  | 89.3 | 88.4 |
|  | Wisconsin | 1,609 |  | 85.1 |  | 96.1 | 85.7 | 85.5 | 96.0 |  | 85.2 | 85.1 |  | 101.5 | 93.8 |

*Source:* Authors’ construction using data from You et al. (2017) and Fritz et al. (2015).

*Notes*: Only the top ten regions (ADM1) of maize production (in terms of quantity) are reported for each country. Percentages over 100 percent are possible due to interventions made to the cropland available to facilitate an entropy solution (see Wood-Sichra et al. 2016 for more details).
