# Supplementary material for: Pixelating crop production: Consequences of methodological choices
Source: PLoS One. 2019 Feb 19;14(2):e0212281. doi: 10.1371/journal.pone.0212281 (PMC6380596; doi:10.1371/journal.pone.0212281)
Supplement: S5 Appendix — (DOCX) [file pone.0212281.s005.docx]

# S5 Appendix

To reduce noise within our original and robustness test estimates, values for the “spatially averaged” estimates are calculated as a linear combination (according to a set of *focal weights*) of pixel values within a defined neighborhood. The focal weights within the neighborhood of any particular pixel $i$ are determined by a 2-dimensional Gaussian filter such that:

(S5-1) $G\left( x, y, \sigma\right)_{i}=\frac{1}{2\pi\sigma^{2}}e^{-\frac{x^{2}+y^{2}}{2\sigma^{2}}}$

where $x$ is the distance from pixel $i$ along the horizontal axis, $y$ is the distance from pixel $i$ along the vertical axis and $\sigma$ is the standard deviation of the Gaussian probability density function. Focal weights are normalized to sum to one within the specified neighborhood.

Fig A, Panel a shows examples of different sets of focal weights (expressed as percentages) using a circular neighborhood of various radii. According to the Gaussian function, the most weight is placed on pixel $i$ (highlighted with a dashed, red line) and successively less weight on more distant pixels within the designated neighborhood (solid, red line). To maintain the shape of the (infinite) Gaussian curve when we use a finite set of focal weights, we choose a value of $\sigma$ such that the radius of the focal weight is equal to 3$\sigma$. This is because 99.7 percent of the total integral of an infinite Gaussian filter falls within a radius of $3\sigma$.

Fig A, Panel b shows cropland in Nigeria that has been smoothed using focal weights with increasing combinations of radius and $\sigma$. As the size of the neighborhood increases, the variation among the now “spatially averaged” pixels decreases. SPAM2005 reports estimates at a 5 arc-minute pixel resolution, which is equal to approximately 10 kilometers at the equator. For the globe, crop producing pixel sizes range from 2,774 hectares to 8,548 hectares. Given this relatively large resolution, it seems unlikely that pixels outside of $3x3$ (1 pixel radius) window would influence the spatial patterns of production within a single pixel. Thus, we use a set of focal weights with a 1 pixel radius and $\sigma=0.33$ to spatially smooth our data.

**Fig A: Differences in cropland extent using Gaussian-based focal weights of varying radii and standard deviation** $\boldsymbol{\sigma}$

| Panel a: Focal weights | | | | |
| --- | --- | --- | --- | --- |
| 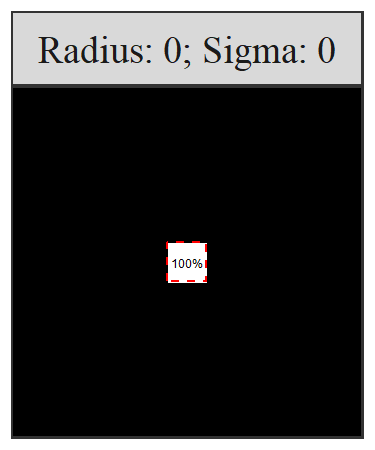 | 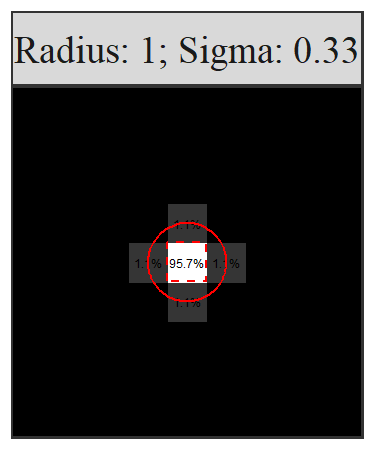 | 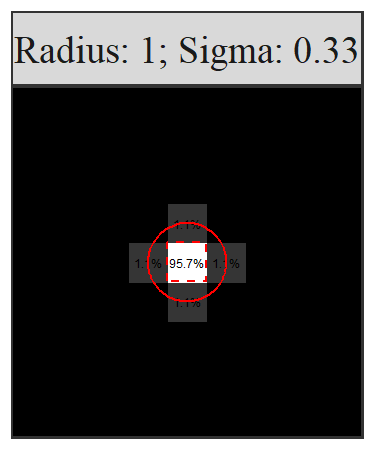 | 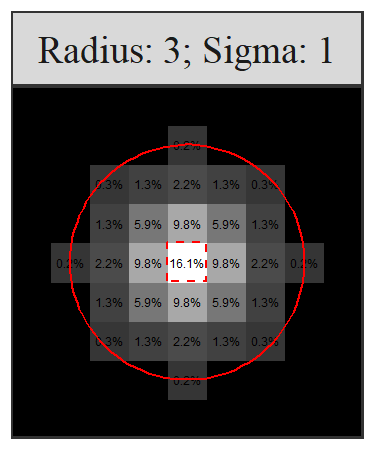 | 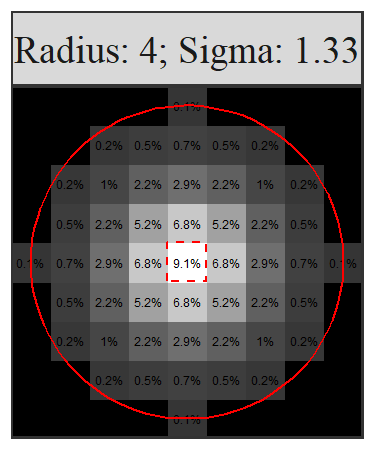 |
| Panel b: Cropland extent in Nigeria | | | | |
| 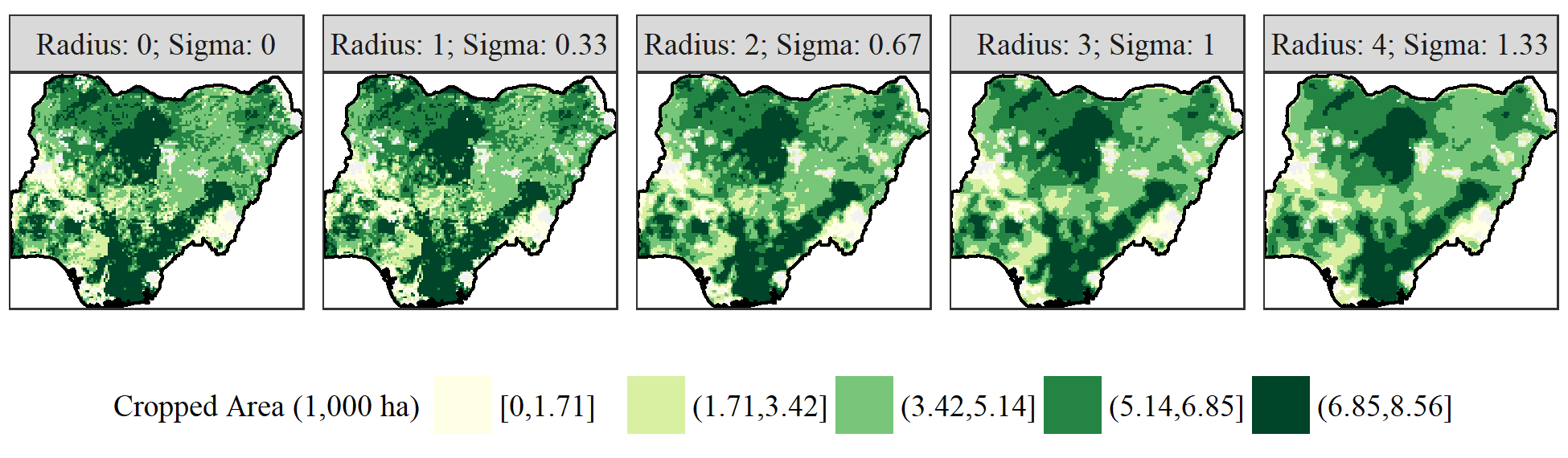 | | | | |

*Source:* Authors’ construction using data from Fritz et al. (2015).

*Note:* The dashed, red line highlights the central pixel of interest in Panel a, while the solid red circle highlights the neighborhood of interest.

## References

Fritz, S., L. See, I. McCallum, L. You, A. Bun, E. Moltchanova, M. Duerauer, F. Albrecht, C. Schill, C. Perger, P. Havlik, A. Mosnier, P. Thornton, U. Wood-Sichra, M. Herrero, I. Becker-Reshef, C. Justice, M. Hansen, P. Gong, S. Abdel Aziz, A. Cipriani, R. Cumani, G. Cecchi, G. Conchedda, S. Ferreira, A. Gomez, M. Haffani, F. Kayitakire, J. Malanding, R. Mueller, T. Newby, A. Nonguierma, A. Olusegun, S. Ortner, D.R. Rajak, J. Rocha, D. Schepaschenko, M. Schepaschenko, A. Terekhov, A. Tiangwa, C. Vancutsem, E. Vintrou, W. Wenbin, M. van der Velde, A. Dunwoody, F. Kraxner and M. Obersteiner. 2015. “Mapping Global Cropland and Field Size.” *Global Change Biology*, 21: 1980–1992. Available from URL: http://www.wur.nl/nl/Publicatie-details.htm?publicationId=publication-way-333433393832 [Accessed December 2016].
