# Supplementary material for: Pixelating crop production: Consequences of methodological choices
Source: PLoS One. 2019 Feb 19;14(2):e0212281. doi: 10.1371/journal.pone.0212281 (PMC6380596; doi:10.1371/journal.pone.0212281)
Supplement: S6 Appendix — (DOCX) [file pone.0212281.s006.docx]

# S6 Appendix

The choice of allocation methodology may have consequences for the pixelated estimates from SPAM2005 that may vary by crop, country, or production statistic. Figs A through I plot heatmaps of $SSI$ (spatial similarity indexes) by crop for each of the nine countries of interest. The crop-level $SSI$ values are averaged over each of the pixel-level $SSI$s within a crop-specific raster for each country and production statistic. In each country, typically only one or two crops dominate production. Crop weights are plotted to the left of the $SSI$ heatmaps in Figs A through I. These weights are used to calculate the country-level $SSI$ values plotted in Fig 1 in the main text. In every country of interest, the crop-level $SSI$ values for predominate crops of production basically mirror the country-level $SSI$ values in Fig 1. These crops are soybeans in Brazil; rice and maize in China; other cereals and maize in Ethiopia; wheat in France; rice in India and Indonesia; sorghum in Nigeria; wheat in Turkey; and soybeans and maize in the United States.

## References

You, L., U. Wood-Sichra, S. Fritz, Z. Guo, L. See, and J. Koo. 2017. Spatial Production Allocation Model (SPAM) 2005 version 3 release 1. *HarvestChoice Data Product*. Washington, D.C.: International Food Policy Research Institute (IFPRI) and St. Paul: International Science and Technology Practice and Policy (InSTePP) Center, University of Minnesota [Accessed May 2017].

**Fig A. Spatial sensitivity of production to each robustness run relative to original estimates in Brazil**


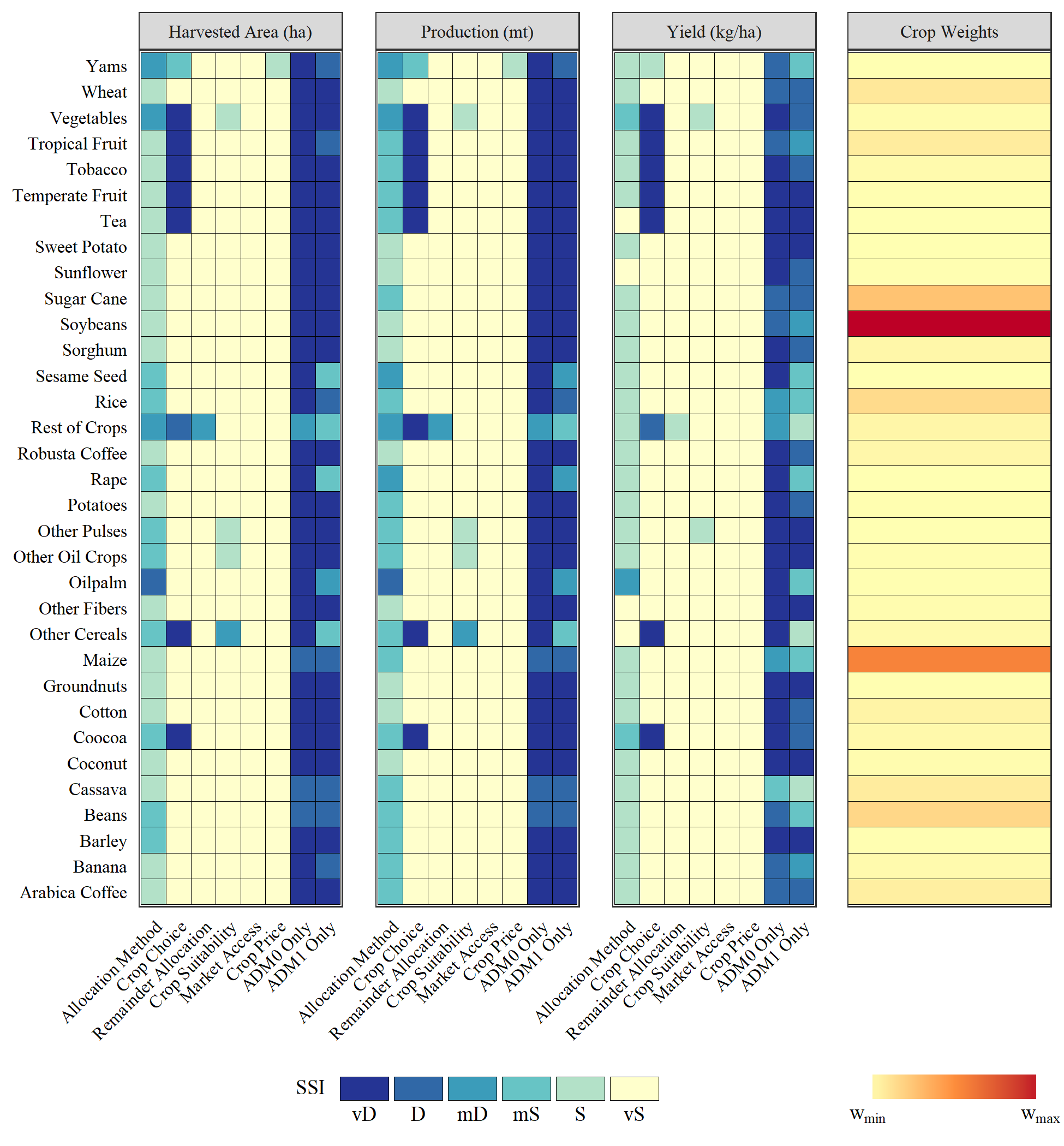


*Source:* Authors’ construction using data from You et al. (2017).

*Notes:* vD – Very Dissimilar; D – Dissimilar; mD – Marginally Dissimilar; mS – Marginally Similar; S – Similar; vS – Very Similar. The fourth column represents the significance of each crop within the country’s production. Crop weights range from minimum share of area harvested (colored in yellow) to the maximum share (colored in dark red). These weights are used to create Fig 1 in the main paper.

**Fig B. Spatial sensitivity of production to each robustness run relative to original estimates in China**


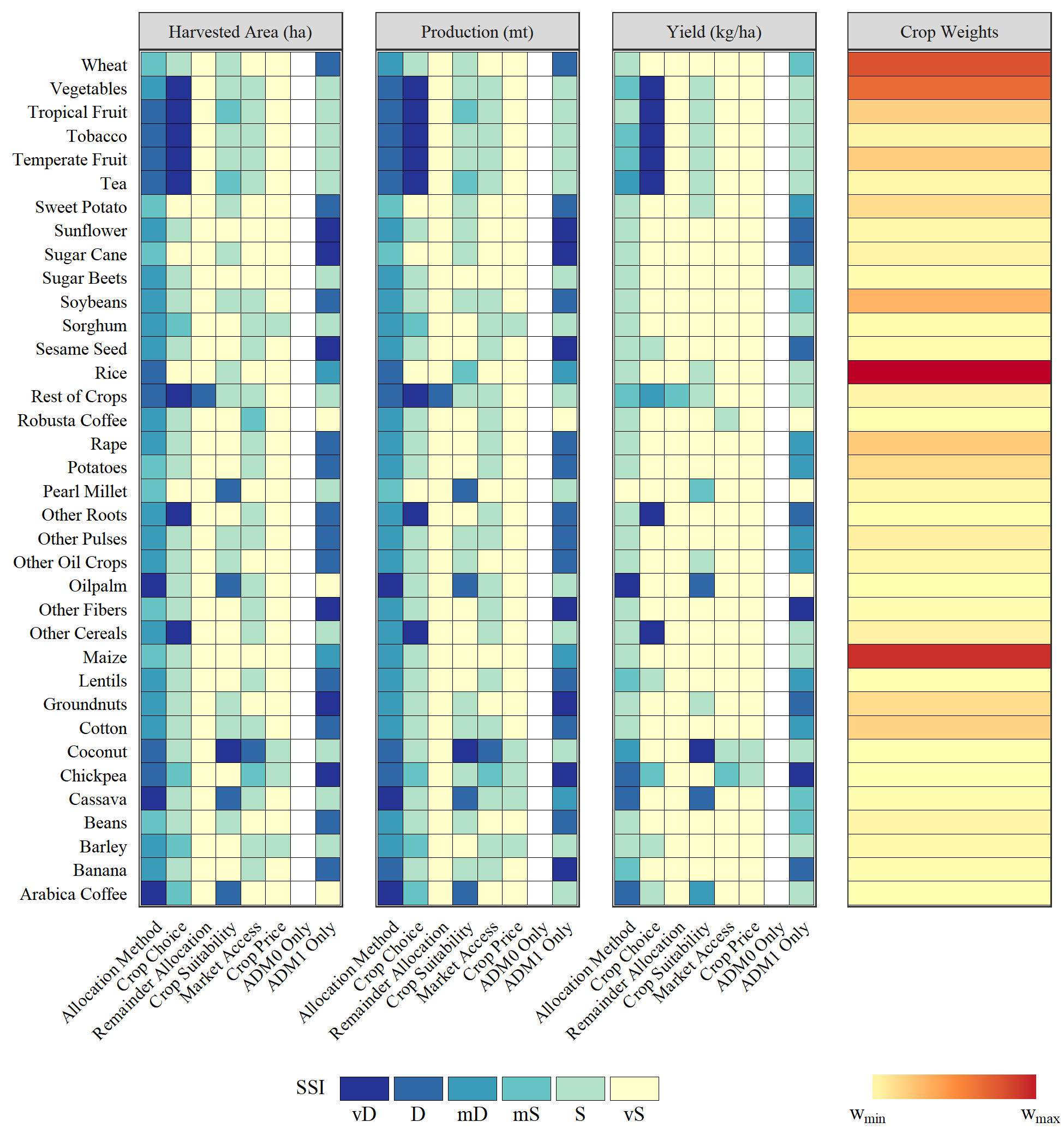


*Source:* Authors’ construction using data from You et al. (2017).

*Notes:* vD – Very Dissimilar; D – Dissimilar; mD – Marginally Dissimilar; mS – Marginally Similar; S – Similar; vS – Very Similar. The fourth column represents the significance of each crop within the country’s production. Crop weights range from minimum share of area harvested (colored in yellow) to the maximum share (colored in dark red). These weights are used to create Fig 1 in the main paper.

**Fig C. Spatial sensitivity of production to each robustness run relative to original estimates in Ethiopia**


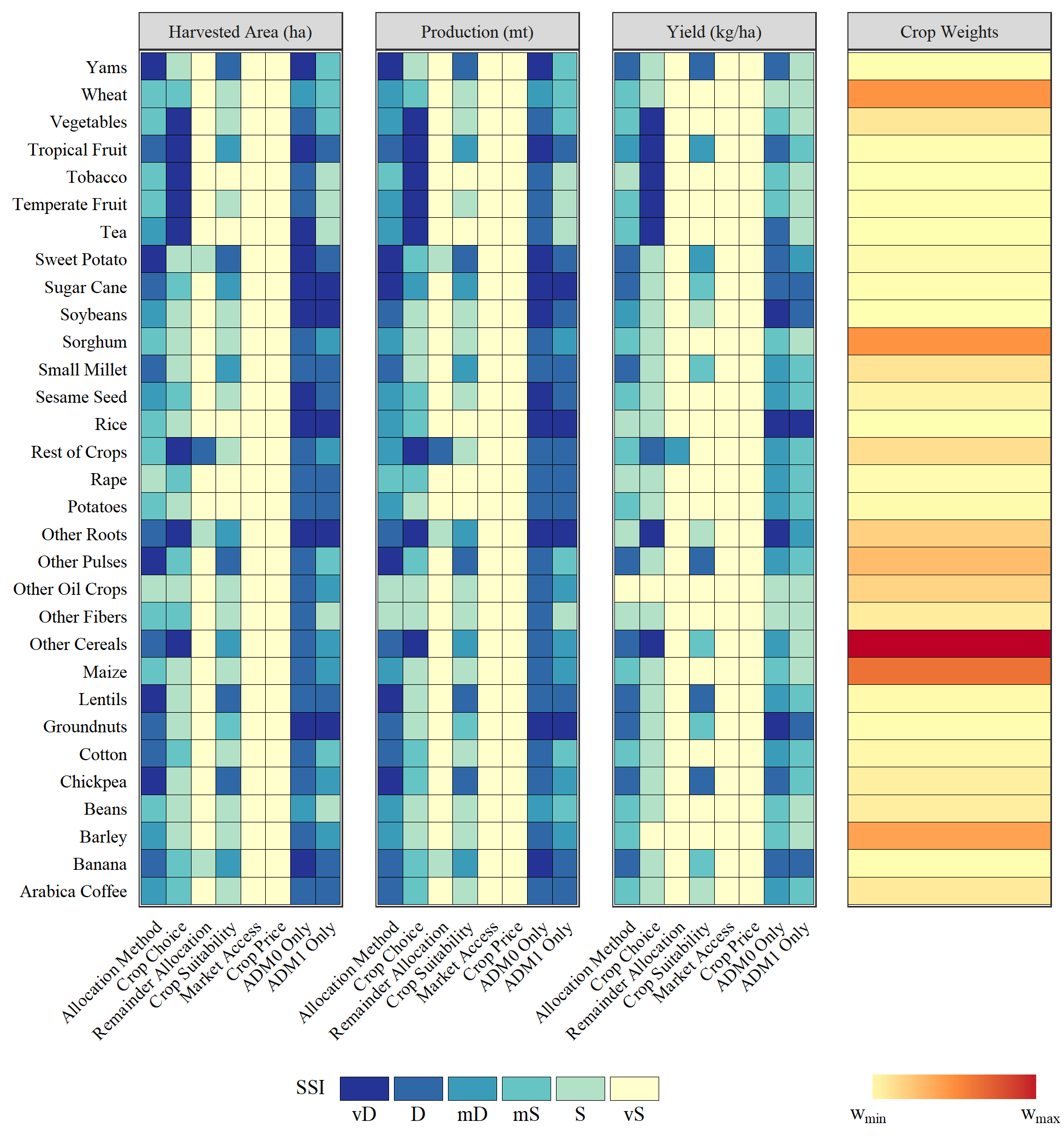


*Source:* Authors’ construction using data from You et al. (2017).

*Notes:* vD – Very Dissimilar; D – Dissimilar; mD – Marginally Dissimilar; mS – Marginally Similar; S – Similar; vS – Very Similar. The fourth column represents the significance of each crop within the country’s production. Crop weights range from minimum share of area harvested (colored in yellow) to the maximum share (colored in dark red). These weights are used create to Fig 1 in the main paper.

**Fig D. Spatial sensitivity of production to each robustness run relative to original estimates in France**


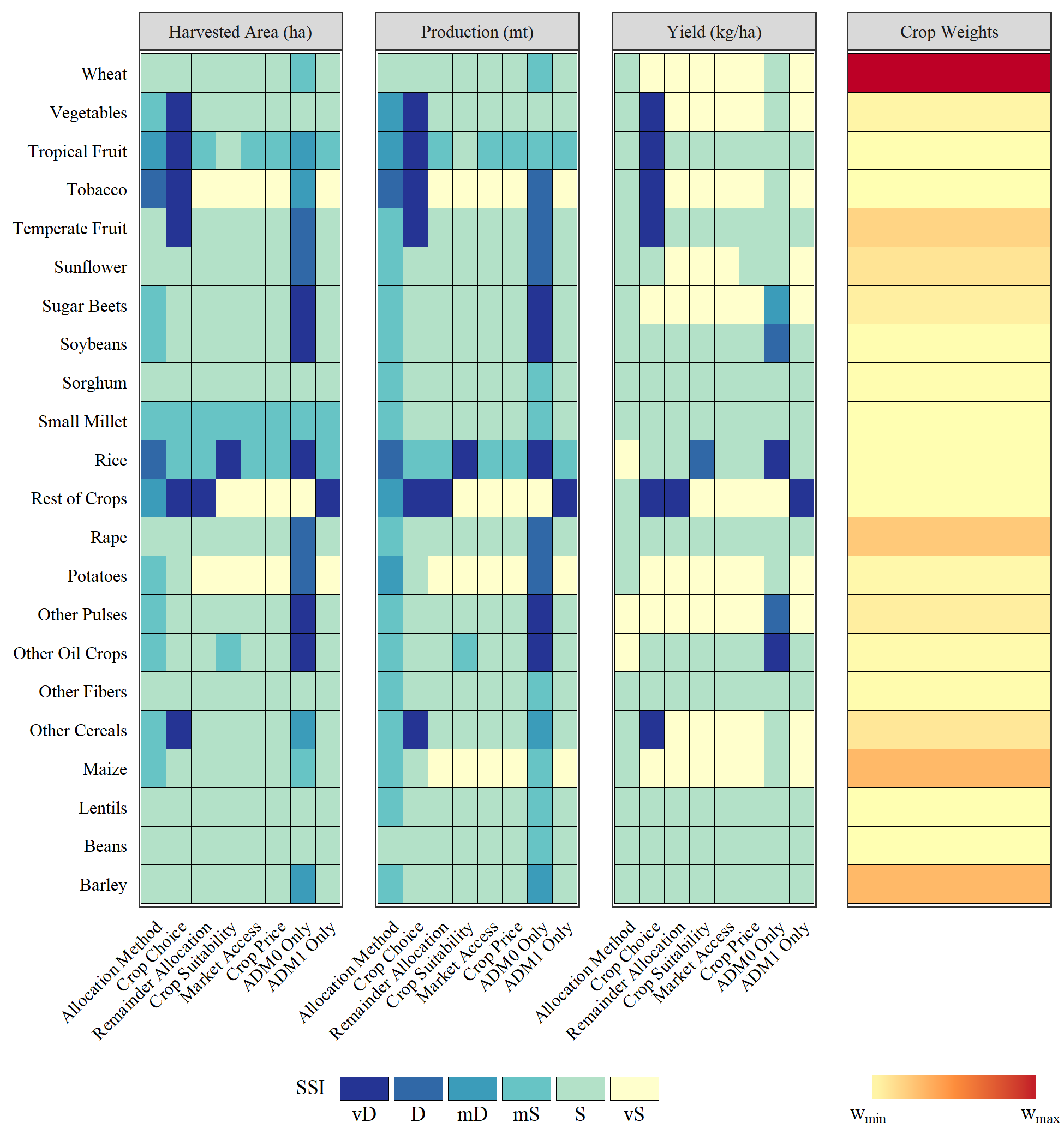


*Source:* Authors’ construction using data from You et al. (2017).

*Notes:* vD – Very Dissimilar; D – Dissimilar; mD – Marginally Dissimilar; mS – Marginally Similar; S – Similar; vS – Very Similar. The fourth column represents the significance of each crop within the country’s production. Crop weights range from minimum share of area harvested (colored in yellow) to the maximum share (colored in dark red). These weights are used to create Fig 1 in the main paper.

**Fig E. Spatial sensitivity of production to each robustness run relative to original estimates in India**


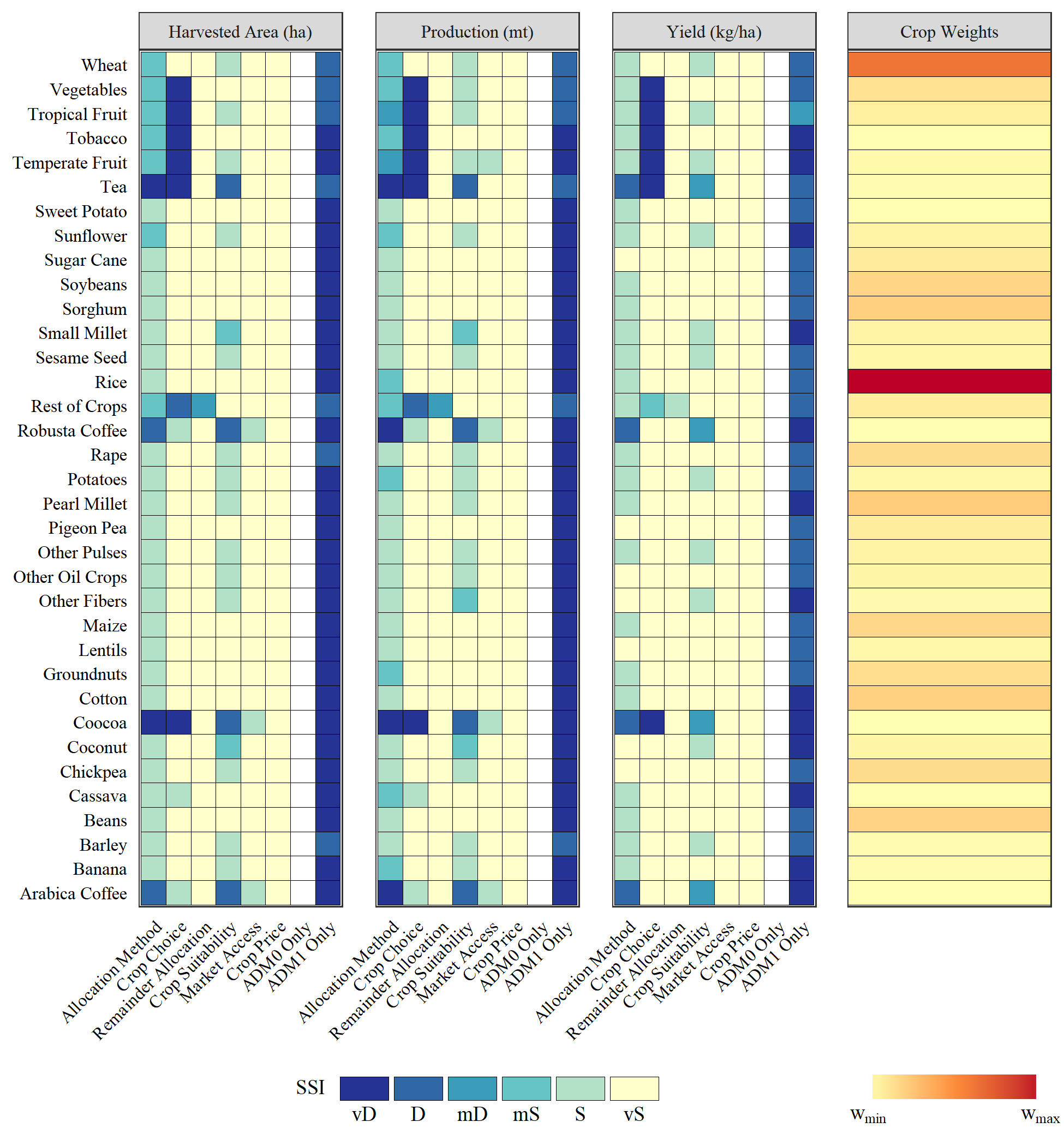


*Source:* Authors’ construction using data from You et al. (2017).

*Notes:* vD – Very Dissimilar; D – Dissimilar; mD – Marginally Dissimilar; mS – Marginally Similar; S – Similar; vS – Very Similar. The fourth column represents the significance of each crop within the country’s production. Crop weights range from minimum share of area harvested (colored in yellow) to the maximum share (colored in dark red). These weights are used to create Fig 1 in the main paper.

**Fig F. Spatial sensitivity of production to each robustness run relative to original estimates in Indonesia**


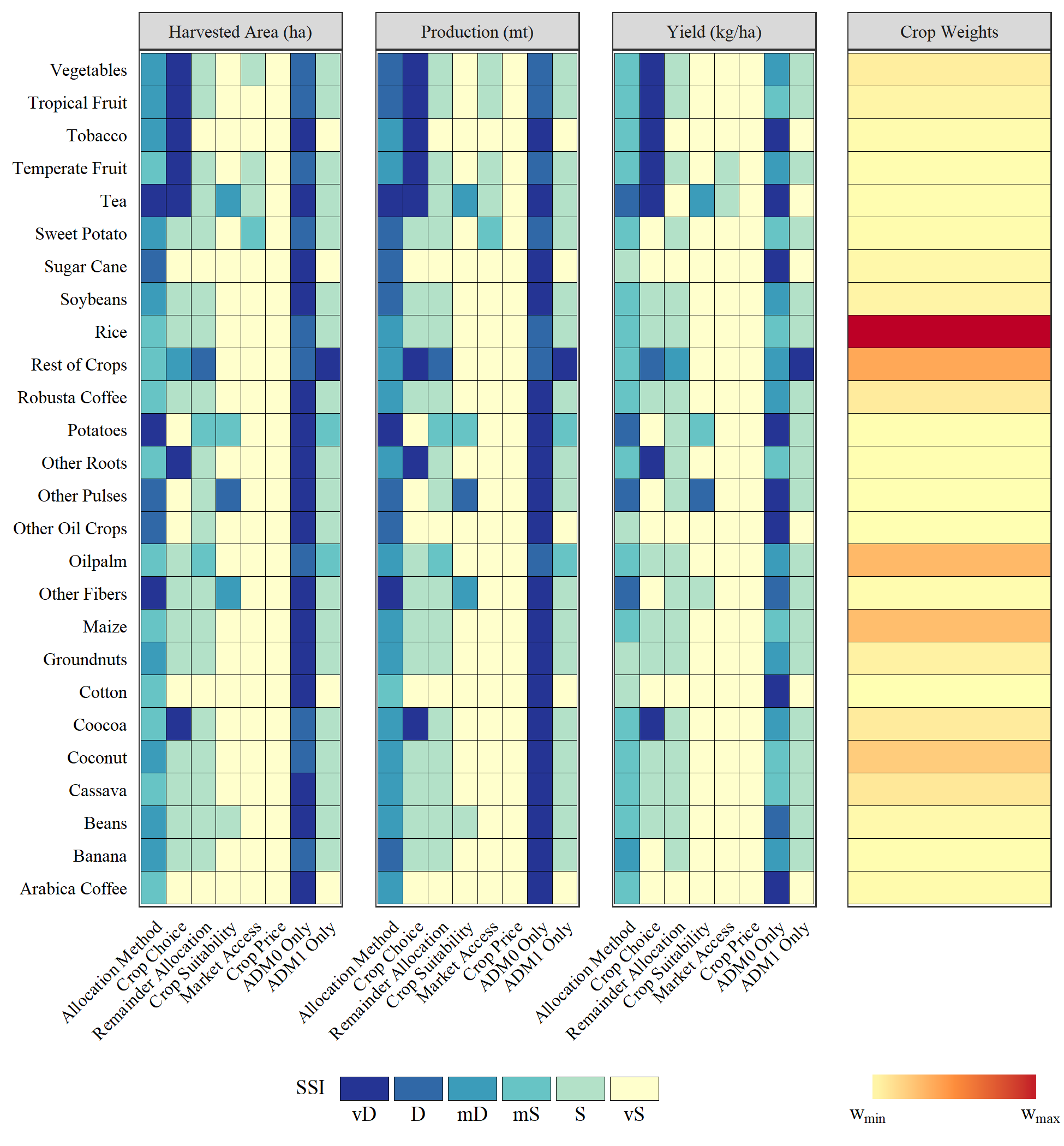


*Source:* Authors’ construction using data from You et al. (2017).

*Notes:* vD – Very Dissimilar; D – Dissimilar; mD – Marginally Dissimilar; mS – Marginally Similar; S – Similar; vS – Very Similar. The fourth column represents the significance of each crop within the country’s production. Crop weights range from minimum share of area harvested (colored in yellow) to the maximum share (colored in dark red). These weights are used to create Fig 1 in the main paper.

**Fig G. Spatial sensitivity of production to each robustness run relative to original estimates in Nigeria**


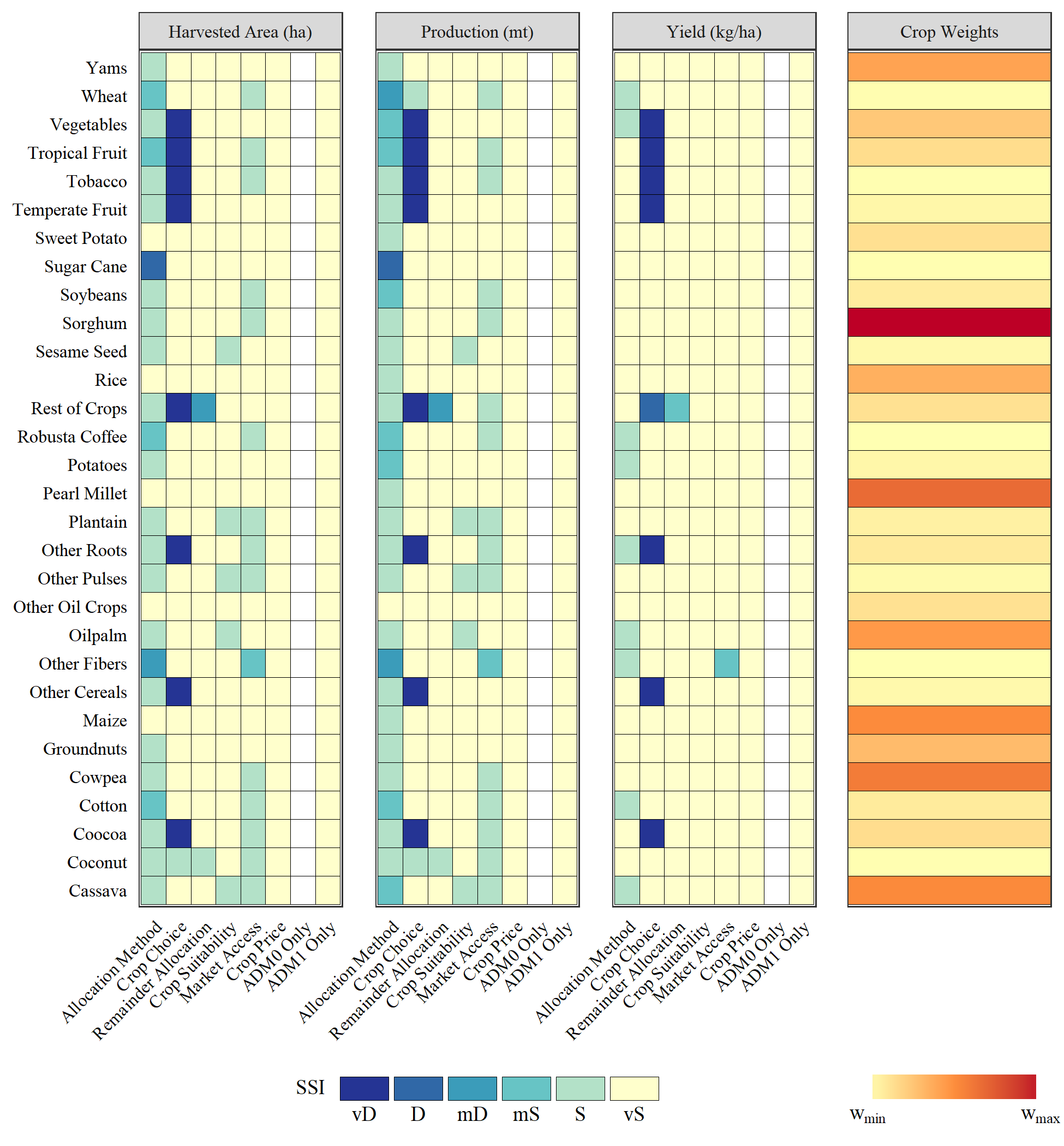


*Source:* Authors’ construction using data from You et al. (2017).

*Notes:* vD – Very Dissimilar; D – Dissimilar; mD – Marginally Dissimilar; mS – Marginally Similar; S – Similar; vS – Very Similar. The fourth column represents the significance of each crop within the country’s production. Crop weights range from minimum share of area harvested (colored in yellow) to the maximum share (colored in dark red). These weights are used to create Fig 1 in the main paper.

**Fig H. Spatial sensitivity of production to each robustness run relative to original estimates in Turkey**


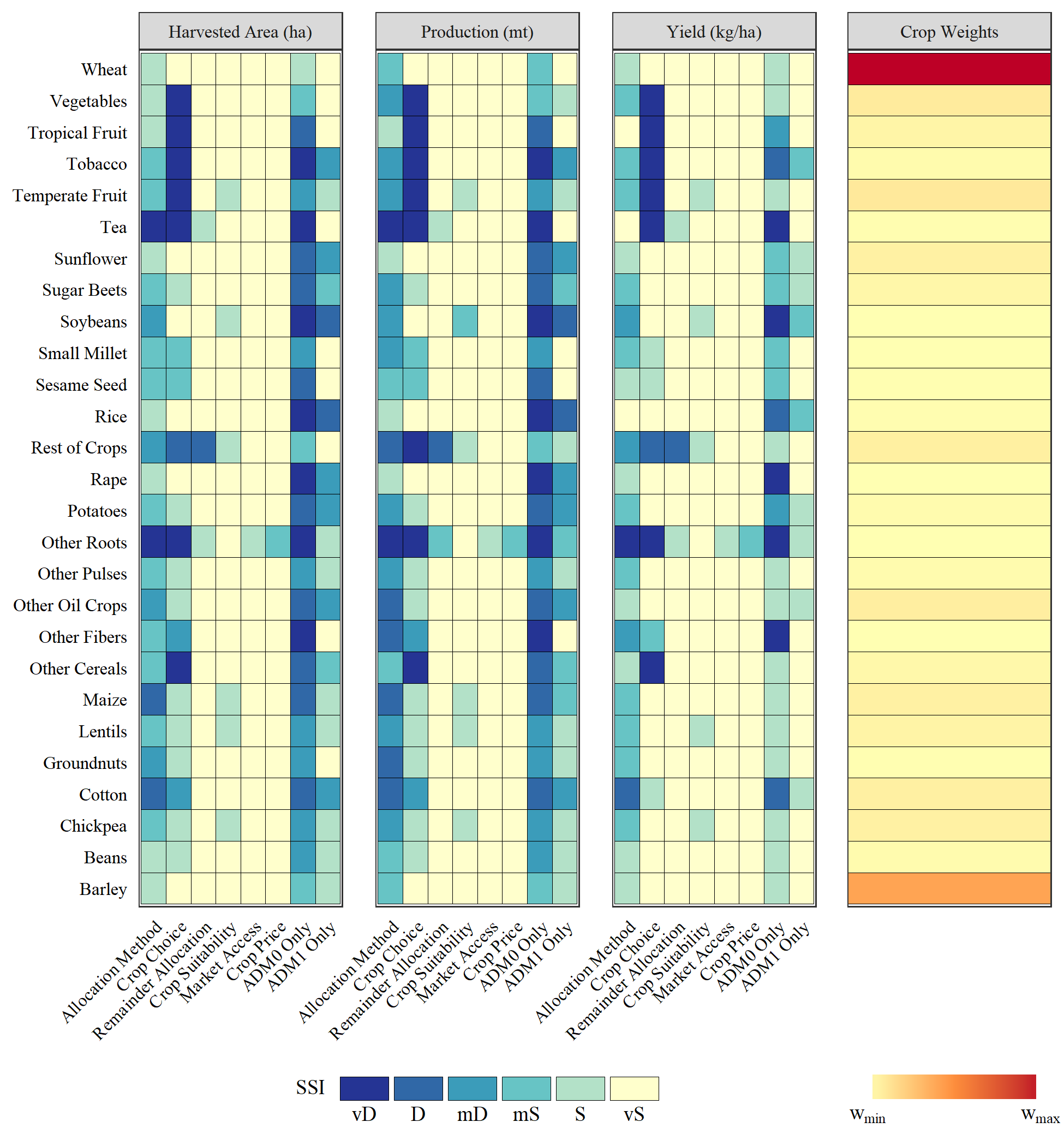


*Source:* Authors’ construction using data from You et al. (2017).

*Notes:* vD – Very Dissimilar; D – Dissimilar; mD – Marginally Dissimilar; mS – Marginally Similar; S – Similar; vS – Very Similar. The fourth column represents the significance of each crop within the country’s production. Crop weights range from minimum share of area harvested (colored in yellow) to the maximum share (colored in dark red). These weights are used to create Fig 1 in the main paper.

**Fig I: Spatial sensitivity of production to each robustness run relative to original estimates in the United States**


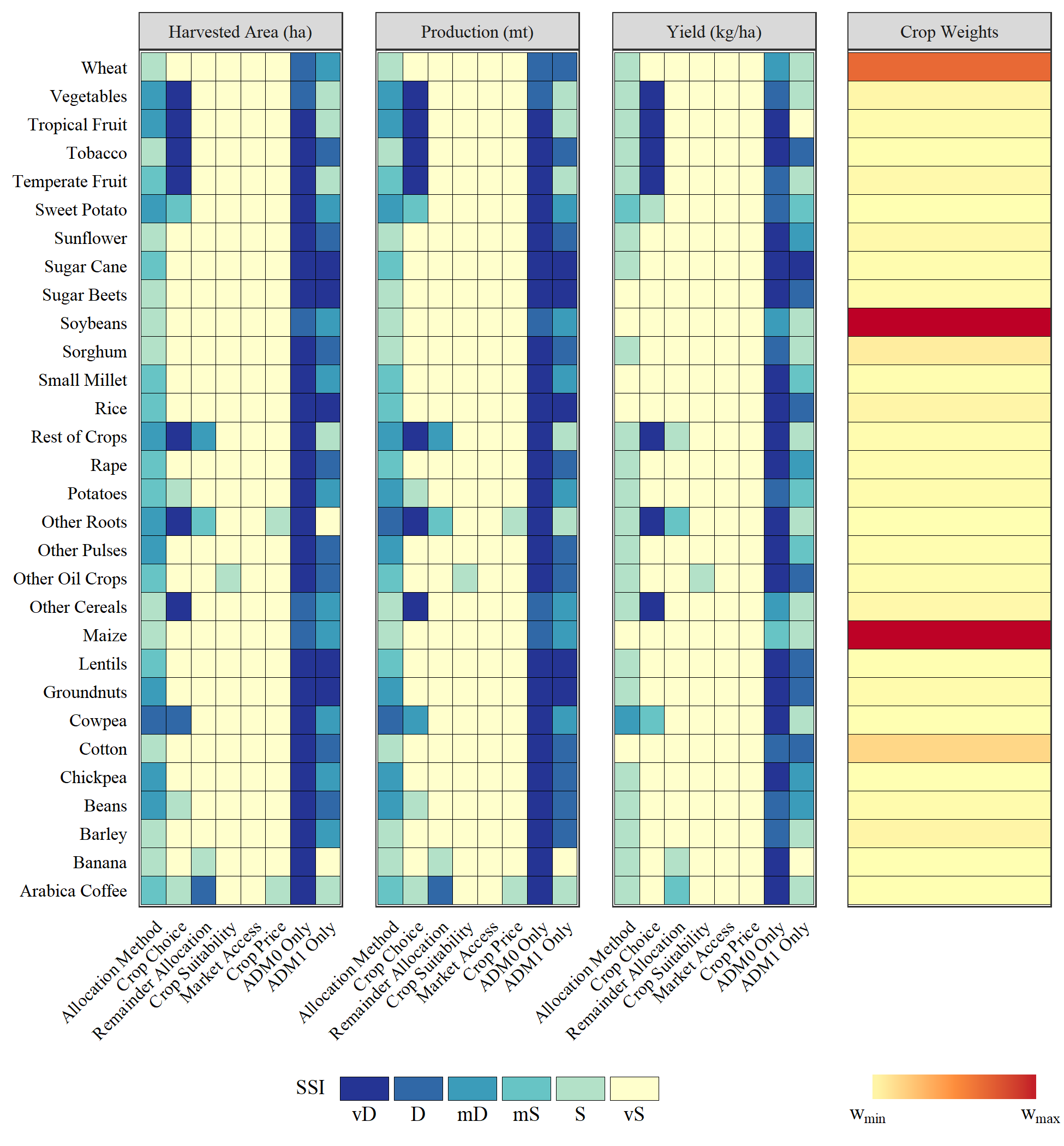


*Source:* Authors’ construction using data from You et al. (2017).

*Notes:* vD – Very Dissimilar; D – Dissimilar; mD – Marginally Dissimilar; mS – Marginally Similar; S – Similar; vS – Very Similar. The fourth column represents the significance of each crop within the country’s production. Crop weights range from minimum share of area harvested (colored in yellow) to the maximum share (colored in dark red). These weights are used to create Fig 1 in the main paper
