# Supplementary material for: Pixelating crop production: Consequences of methodological choices
Source: PLoS One. 2019 Feb 19;14(2):e0212281. doi: 10.1371/journal.pone.0212281 (PMC6380596; doi:10.1371/journal.pone.0212281)
Supplement: S7 Appendix — (DOCX) [file pone.0212281.s007.docx]

# S7 Appendix

To examine differences in the (global) spatial autocorrelation between the original SPAM2005 estimates and each set of estimates arising from our robustness runs, we calculated Moran’s I statistic for each crop and country combination. Moran’s I statistic ranges from -1 (complete dispersion) to 1 (complete concentration), where a value of zero represents a random pattern of production. This statistic simultaneously accounts for both the location and amount of production. Fig A is a scatterplot of Moran’s I statistics from the original run and the robustness runs for each country of interest for harvested area. Points are colored by robustness scenario and their shape denotes one of four major crops: maize, rice, sorghum or wheat. While there are differences in the level of spatial autocorrelation by robustness scenario and crop, there are not obvious differences between the estimates from the original run and those from the robustness runs. For each country, points fall on or near the forty-five degree line (black dotted line). Thus, it does not appear that any of the methodological cum data choices in question result in different clustering patterns of production. Similar conclusions hold for production quantity and yield.

## References

You, L., U. Wood-Sichra, S. Fritz, Z. Guo, L. See, and J. Koo. 2017. Spatial Production Allocation Model (SPAM) 2005 version 3 release 1. *HarvestChoice Data Product*. Washington, D.C.: International Food Policy Research Institute (IFPRI) and St. Paul: International Science and Technology Practice and Policy (InSTePP) Center, University of Minnesota [Accessed May 2017].

**Fig A: Comparison of spatial autocorrelation between robustness runs and original estimates, harvested area**


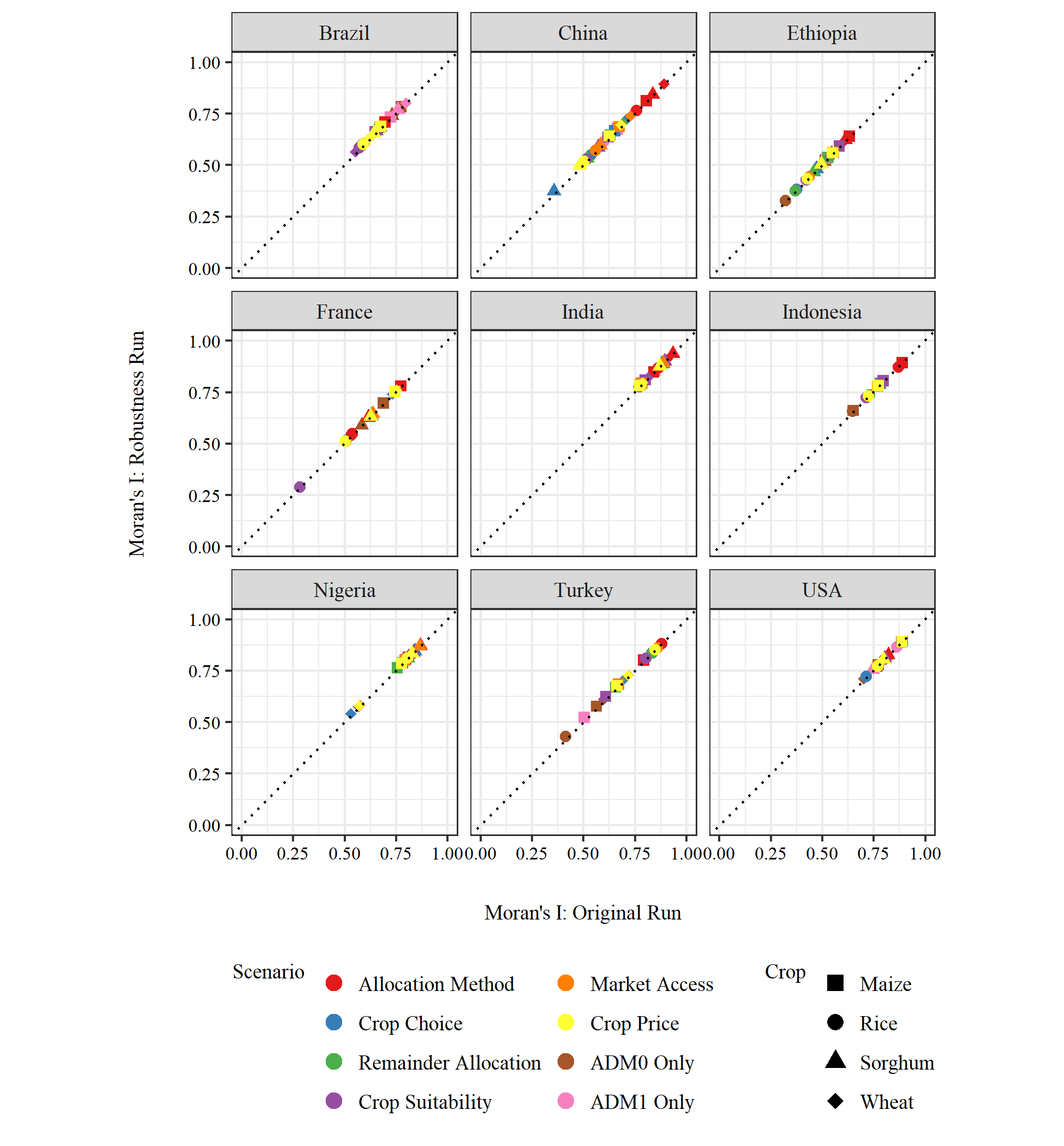


*Source:* Authors’ construction using data from You et al. (2017).

*Notes:* The forty-five degree line is represented by the dotted black line.
